# Smooth muscle-specific expression of hydroxyindole O-methyltransferase reduces arterial injury-induced intimal hyperplasia

**DOI:** 10.1186/s12929-025-01172-4

**Published:** 2025-08-20

**Authors:** Wei-Cheng Jiang, Chung-Huang Chen, Hua-Hui Ho, Pei-Yu Gung, Jing-Yiing Wu, Cheng-Chin Kuo, Kenneth K. Wu, Shaw-Fang Yet

**Affiliations:** 1 Institute of Anatomy and Cell Biology, College of Medicine, National Yang Ming Chiao Tung University, Taipei, 112304 Taiwan; 2 Institute of Cellular and System Medicine, National Health Research Institutes, Zhunan, 350401 Taiwan

**Keywords:** Hydroxyindole O-methyltransferase, 5-Methoxytryptophan, Serotonin, Intimal hyperplasia, Vascular smooth muscle cells

## Abstract

**Background:**

The pineal gland produces melatonin to control circadian rhythm via the final enzyme in the serotonin pathway, hydroxyindole O-methyltransferase (HIOMT). Interestingly, HIOMT is expressed by certain non-pineal cells. The main catalytically active of the three human HIOMT (hHIOMT) isoforms in pineal cells is hHIOMT345 (345 amino acids), while hHIOMT298 (298 amino acids) is the most active isoform in fibroblasts, where it converts 5-hydroxytryptophan to 5-methoxytryptophan (5-MTP). We previously demonstrated that exogenous 5-MTP protects the arteries. Nevertheless, whether vascular smooth muscle cells (VSMCs) per se synthesize 5-MTP is unknown.

**Methods:**

We transfected primary wild-type VSMCs with different *hHIOMT* isoforms and treated them with inflammatory cytokines to examine *hHIOMT’s* effects on p38 MAPK activation. Global and VSMC-specific *hHIOMT* transgenic mice were generated and subjected to an arterial injury model. Histological analysis was performed to evaluate intimal hyperplasia and expression of select tryptophan metabolites and their synthetic enzymes. We treated wild-type and transgenic VSMCs with interleukin-1 beta (IL-1β), with or without 5-MTP, to examine the levels of serotonin and aromatic L-amino acid decarboxylase (AADC). Serotonin’s effects on VSMC functions were evaluated, and inhibitors of p38 MAPK and ERK1/2 were used to determine the signaling pathways. The effects of AADC on VSMCs were assessed by AADC knockdown or overexpression.

**Results:**

Overexpression of the human full-length isoform of 373 amino acids (hHIOMT373) in VSMCs attenuated proinflammatory cytokine-induced p38 MAPK activation, similar to 5-MTP treatment. Global and VSMC-specific hHIOMT373 transgenic mice exhibited attenuated intimal hyperplasia following arterial injury. Intriguingly, the tryptophan metabolite serotonin and its synthetic enzyme AADC were reduced in transgenic arteries. In VSMCs, IL-1β increased AADC and serotonin levels that were mitigated by 5-MTP treatment or HIOMT overexpression via suppressing the p38 MAPK pathway. Interestingly, serotonin promoted VSMC proliferation and decreased VSMC marker levels through ERK1/2 activation. While AADC overexpression decreased VSMC contractile markers, AADC knockdown suppressed IL-1β-induced VSMC proliferation.

**Conclusions:**

Our results unveiled a unique function of HIOMT in vascular disease. In VSMCs, hHIOMT373 reprogrammed tryptophan metabolism to increase 5-MTP and decrease serotonin levels, thereby protecting against injury-induced intimal hyperplasia. Mechanistically, HIOMT-5-MTP suppressed AADC-serotonin induction through inhibiting p38 MAPK activation.

**Supplementary Information:**

The online version contains supplementary material available at 10.1186/s12929-025-01172-4.

## Background

Hydroxyindole O-methyltransferase (HIOMT, also known as N-acetylserotonin O-methyltransferase, ASMT), initially reported to be specifically expressed in the pineal gland and retina [1], is the last enzyme of melatonin biosynthesis in the serotonin (also known as 5-hydroxytryptamine) pathway by converting N-acetylserotonin to melatonin [2]. Intriguingly, in addition to pineal cells, HIOMT was found in non-pineal cells and catalyzes the production of 5-methoxytryptophan (5-MTP) from 5-hydroxytryptophan in fibroblasts [3], representing a new branch of the serotonin pathway [4] and suggesting versatile biological roles of HIOMT. Human HIOMT (hHIOMT) has 3 isoforms: a full-length 373 amino acids (hHIOMT373), a 345 amino acid protein without exon 6 (hHIOMT345), and a 298 amino acid protein lacking exon 6 and 7 (hHIOMT298) [5]. hHIOMT345 is identified as the major catalytically active HIOMT isoform in human pineal cells [2], while the predominant HIOMT isoform expressed in non-pineal cells is hHIOMT298 [6]. Interestingly, forced expression of hHIOMT298 in cancer cells attenuates cancer progression and suppresses aromatic L-amino acid decarboxylase (AADC; also known as dopa decarboxylase) expression that converts 5-hydroxytryptophan to serotonin [6], suggesting intricate interplays among tryptophan metabolic pathways.

Tryptophan is an essential amino acid, and its many metabolites contribute to the regulation of human health and disease [7]. Abnormal tryptophan metabolism has been associated with an increased risk of developing autoimmune, inflammatory, cardiovascular, and oncological diseases [8]. Tryptophan is catabolized via two main pathways: ~ 95% of tryptophan is converted to kynurenine via the kynurenine pathway, whereas ~ 5% of tryptophan is metabolized via the serotonin pathway, converting 5-hydroxytryptophan to serotonin and subsequently to melatonin [9]. Serotonin is a critical neurotransmitter in the central nervous system and modulates neural activity and neuropsychological processes; serotonergic dysregulation leads to various neuropsychiatric and neurological disorders [10]. Besides critical functions in the central nervous system, serotonin has expanding roles in the peripheral system [11]. Though serotonin is an essential neurotransmitter, peripheral serotonin is associated with cardiovascular disease risk and can produce harmful effects, such as vascular resistance, pulmonary hypertension, coronary artery disease, and the occurrence of cardiac events [11–13]. Many kynurenine pathway-derived metabolites, particularly kynurenine, promote inflammation and are related to inflammatory diseases [4, 14]. A number of cardiovascular diseases are associated with the overactivation of the kynurenine pathway [4, 15].

Although some tryptophan metabolites exert pathological effects, certain metabolites have important physiological and/or protective functions. 5-MTP was initially identified in the rat pineal gland to have a possible physiological role in reproduction [16]. It was later discovered that normal fibroblasts can produce and release 5-MTP into the extracellular milieu to protect against cancer growth by suppressing inflammatory cyclooxygenase-2 expression [3]. Further studies showed that 5-MTP can also be derived from endothelial cells and defends against endothelial barrier dysfunction and excessive systemic inflammatory responses mainly through the p38 MAPK pathway [17, 18]. One study showed that 5-MTP levels are inversely correlated with clinical markers of kidney disease [19]. Another study revealed that 5-MTP attenuates postinfarct cardiac injury by controlling oxidative stress and immune activation in rats [20]. Furthermore, plasma 5-MTP was reported to be an independent and protective early biomarker for major adverse cardiovascular and heart failure events in patients with acute myocardial infarction [21]. Together, these studies indicate an emerging anti-inflammatory, protective function of 5-MTP in cells and tissues.

We have previously demonstrated a protective role of 5-MTP in occlusive vascular disease by reducing intimal hyperplasia following arterial injury [22, 23]. 5-MTP decreases inflammatory cell infiltration, accelerates endothelial recovery, and inhibits vascular smooth muscle cell (VSMC) proliferation and migration into intimal space. The vascular protective function of 5-MTP was further supported by a study showing that 5-MTP protects against atherosclerosis and calcification by inhibiting TLR2-mediated VSMC phenotypic switch to chondrocytes and the consequent calcification [24]. Taken together, 5-MTP clearly plays critical functions in VSMCs. However, it is not clear whether VSMCs per se produce 5-MTP.

In the present study, we showed that of the three hHIOMT isoforms, hHIOMT373 was the active isoform in VSMCs and functioned to protect against injury-induced vessel occlusion via reprogramming tryptophan metabolism by increasing 5-MTP and decreasing serotonin. HIOMT/5-MTP abrogated serotonin and its synthetic enzyme AADC expression by suppressing p38 MAPK signaling, whereas serotonin exerted opposing effects of 5-MTP in VSMCs via the ERK1/2 pathway. Most significantly, our study uncovered a previously unrecognized function of HIOMT in vascular disease.

## Materials and methods

### Expression plasmids

The open reading frame of the mouse *Hiomt* cDNA from the C3H strain that encodes the wild-type *Hiomt* sequence (NCBI reference sequence: NM_001199212.1) or the C57BL/6 strain that contains 2 mutations (R78G and R242C) at the catalytic site (GenBank: AB512670.1) with impaired catalytic activity [25] was commercially synthesized and cloned into the pUC57 vector (Omics Bio, New Taipei City, Taiwan). The cDNA fragments were subsequently excised and cloned into the Hind III and BamH I restriction enzyme sites of the pFlag-CMV vector (Sigma- Aldrich, St. Louis, MO, USA; E7523) to generate pFlag-CMV-*mHiomt*-C3H and pFlag-CMV-*mHiomt*-C57BL expression plasmids, respectively. The human *HIOMT* expression plasmids were purchased from GenScript (Piscataway, NJ, USA): the pcDNA3.1 + C-(K)-DYK-*hHIOMT373* plasmid contained the full length(1122 bp, 373 amino acids) *hHIOMT373* (CloneID OHu19659)*,* and the pcDNA3.1 + C-(K)-DYK-*hHIOMT298* plasmid contained a variant (897 bp, 298 amino acids, lacking amino acids 189–235) of *hHIOMT* (CloneID OHu17467). The human *AADC* (*hAADC*) expression plasmid pCMV6-AC-GFP-*hAADC* (RG201345) and pCMV6-AC-GFP vector (PS100010) were purchased from OriGene Technologies (Rockville, MD, USA). To generate the transgenic construct pCAG-*hHIOMT373* expressing *hHIOMT373* under the control of CAG (CMV enhancer/chicken β-actin promoter/splice acceptor of the rabbit β-globin gene), the HcRed fragment of the pCAG-HcRed plasmid [26] (Addgene, Watertown, MA, USA; #11152) was excised and replaced with the DYK (Flag)-*hHIOMT373* fragment (between Nhe I and Not I restriction enzyme sites) from pcDNA3.1 + C-(K)-DYK-*hHIOMT373*..

### Mouse VSMC primary culture and transient transfection assays

Primary VSMCs were isolated from wild-type and *hHIOMT373* transgenic mouse aortas of the C57BL/6J strain and cultured in DMEM containing 10% FBS, and passage 5–7 cells were used for experiments [27]. For overexpression experiments, 1 × 10^6^ VSMCs in 100 µL of electroporation buffer (BIO-RAD, Hercules, CA, USA; #1652677) were electroporated with 8 µg of vector or expression plasmid with the Gene Pulser Xcell Electroporation system (Bio-Rad; #1652660) with a single pulse of square wave 400V for 10 ms). Following recovery, cells were serum starved in 0.2% FBS medium, treated with or without inflammatory cytokine, and protein isolated at indicated time points for Western blot analysis. To assess the expression of the exogenous hHIOMT373 by the transgenic construct pCAG-*hHIOMT373* in VSMCs, wild-type cells were transfected with the pCAG empty vector or pCAG-*hHIOMT373* plasmid and then plated on a 60-mm dish for protein isolation 24 h later for Western blot analysis using an anti-hHIOMT antibody (Abcam; ab180511) to detect hHIOMT.

### siRNA knockdown

To suppress mRNA levels of *AADC* in the mouse wild-type VSMCs, we performed knockdown experiments with small interfering RNA (siRNA). The ON-TARGET*plus* SMART pool mouse *AADC* siRNA (L-065103–01-0010) and negative control siRNA, ON-TARGET*plus* Non-targeting Control Pool (D-001810–10), were obtained from Dharmacon (Lafayette, CO, USA). VSMCs (2.8 × 10^5^ cells) were plated on a 60-mm dish, and after overnight incubation, cells were transfected with 20 nmol/L of negative control or *AADC* siRNA in Opti-MEM® I Reduced Serum Medium (Gibco, Waltham, MA, USA; #31985062) using Lipofectamine RNAiMAX Transfection Reagent (Invitrogen, Waltham, MA, USA; #13778150) as described by the manufacturer’s protocol. Following overnight incubation, cells were serum-starved and then treated with 25 µmol/L of serotonin (Sigma-Aldrich; H9523) and/or 10 ng/mL of interleukin-1 beta (IL-1β) (PeproTech, Cranbury, NJ, USA; #211-11B) for 24 h. Cellular proliferation was then measured with Cell Counting Kit-8 (CCK-8, Dojindo Molecular Technologies, Rockville, MD, USA; CK04-01) assays.

### Western blot analysis

Total proteins were prepared from control or treated wild-type or *hHIOMT373* transgenic VSMCs and subjected to SDS polyacrylamide gel electrophoresis. Proteins were then transferred to PVDF membranes (Merck Millipore, Danvers, MA, USA; #88518) for Western blot analysis. To examine the contents within the medium that may contain 5-MTP to affect VSMC function, the conditioned medium (CM) from vector- or *hHIOMT373*-transfected VSMCs was collected and used to treat VSMCs. Western membranes were incubated with primary hHIOMT antibody (Abcam, Waltham, MA, USA; ab180511) to detect expression of hHIOMT373 and hHIOMT298, while Flag antibody (Cell Signaling; Danvers, MA, USA; #14793) was used to detect Flag-mouse HIOMT (mHIOMT-C3H and mHIOMT-C57BL). To detect SMC markers, blots were incubated with primary antibodies against SM 22α (smooth muscle 22 alpha) (Abcam; ab14106), SM α-actin (smooth muscle alpha-actin) (Sigma-Aldrich; A5228), and SM-MHC (smooth muscle myosin heavy chain) (Proteintech, Rosemont, IL, USA; 21404–1-AP). AADC was detected by incubating with AADC primary antibody (Abcam; ab3905). To detect activation of p38 MAPK signaling pathways, blots were hybridized with antibodies for total and phosphorylated p38 (Cell Signaling; #9212 and #9211, respectively), ERK1/2 (Cell Signaling; #9102 and #9106, respectively), and NFκB (nuclear factor kappa B)/p65 (Cell Signaling; #6596 and #3033, respectively). α-Tubulin antibody (Cell Signaling; #3873) was used to verify equal loading. After incubation with the primary antibody, the blots were incubated with the secondary antibody at room temperature for 2 h. The blots were then developed with chemiluminescent HRP substrate and then visualized by exposure to film. The protein bands were quantified by ImageJ 1.53i software and normalized to controls. The antibodies used are described in Additional file 1.

### Immunofluorescence staining

To assess the expression of the *hHIOMT373* transgene and production of 5-MTP of the transgenic construct pCAG-*hHIOMT373* in VSMCs, wild-type cells were transfected with the pCAG empty vector or pCAG-*hHIOMT373* plasmid and then plated on a 6-channel µ-Slide VI (ibidi, Fitchburg, WI, USA; #80601) in triplicate. Twenty-four hours later, the transfected cells were fixed with 2% paraformaldehyde (Santa Cruz Biotechnology, Santa Cruz, CA, USA; sc-281692) and permeabilized with 0.05% Triton X-100 (Sigma-Aldrich; X-100). Following blocking with 1% bovine serum albumin (BSA) (Sigma-Aldrich; A7906) in PBS, cells were incubated sequentially with primary and secondary antibodies. Cells were first incubated with hHIOMT antibody (Abcam; ab180511) at 4 °C overnight, followed by incubation with secondary donkey anti-rabbit IgG (H + L), Alexa Fluor™ 594 (red fluorescence) antibody (Invitrogen; A21207) at room temperature for 1 h. After washing, the cells were incubated with blocking buffer and then incubated with anti-5-MTP antibody (GenScript; custom-made [24]) overnight at 4 °C. The cells were then incubated with a donkey anti-rabbit IgG (H + L), Alexa Fluor™ 488 secondary antibody (Invitrogen; A21206) at room temperature for 1 h to detect 5-MTP (green fluorescence). The cells were subsequently counterstained with DAPI (Sigma-Aldrich; D9542) to stain the nuclei blue and then mounted using a 0.5% fluorescence mounting medium (Dako, Glostrup, Denmark; S3023). Cells were viewed and imaged using a fluorescence microscope (Olympus, Tokyo, Japan; Olympus IX71).

### VSMC proliferation and migration assay

To assess VSMC proliferation, cells were seeded in 96-well plates (5 × 10^3^ cells/well) in triplicate, serum-starved, treated with 10 ng/mL of platelet-derived growth factor-BB (PDGF-BB) (Peprotech; #315–18), IL-1β, or tumor necrosis factor-alpha (TNF-α) (Prospec, East Brunswick, NJ, USA; CYT-252) for 24 h, and then proliferation was measured by CCK-8 assays. To assess VSMC migration, wound healing assays were performed. Briefly, cells were seeded in 6-well plates (5 × 10^5^ cells/well) for 16 h, followed by treating cells with 10 µg/mL mitomycin C (Sigma-Aldrich; M4287) for 2 h to arrest growth and then wounding with a p200 tip. Cells were then treated with vehicle, 10 ng/mL of PDGF-BB, IL-1β, or TNF-α in starvation medium for 6 h. Wound images were captured at time 0 and 6 h later. Wound closure was quantified by ImageJ 1.53i software from 3 fields of each treatment.

### Effect of serotonin on VSMC proliferation, migration, and marker expression

To examine the effect of serotonin on VSMC proliferation, serum-starved wild-type and/or *hHIOMT373* transgenic VSMCs were treated with different concentrations (0, 5, 10, 25, and 50 µmol/L) of serotonin (Sigma-Aldrich; H9523) in the presence or absence of IL-1β for 24 h, and proliferation was measured. To assess the effect of serotonin on VSMC migration, serum-starved wild-type VSMCs were treated with serotonin (0, 25, and 50 µmol/L) and subjected to wound healing assays to measure migratory capacity. To assess the effect of serotonin on SMC marker expression, serum-starved wild-type VSMCs were treated with or without serotonin (25 µmol/L), in the presence or absence of 5-MTP for 24 h. Total proteins were then prepared for Western blot analysis to detect SM-MHC, SM22α, and SM α-actin expression levels.

### Signaling pathway analysis

To evaluate the effect of 5-MTP or HIOMT overexpression on p38 or NF-κB activation induced by inflammatory cytokines (IL-1β or TNF-α), wild-type VSMCs were electroporated with the vector (pFlag-CMV), *hHIOMT* expression plasmids (pcDNA3.1 + C-(K)-DYK-*hHIOMT373* or pcDNA3.1 + C-(K)-DYK-*hHIOMT298*), or *mHIOMT* expression plasmids (pFlag-CMV-*mHiomt*-C3H or pFlag-CMV-*mHiom*t-C57BL). Following overnight recovery, cells were serum starved for 48 h and then stimulated with 10 ng/mL IL-1β or TNF-α for 15 min. Total proteins were then isolated for Western blot analysis to detect phosphorylation of p38 MAPK and NFκB-p65 (Ser536). To examine the effect of serotonin on p38 and ERK1/2 activation, wild-type and *hHIOMT373* transgenic VSMCs were serum starved and treated with or without serotonin (Sigma-Aldrich; H9523) for 15 min. Proteins were then isolated for Western blot analysis to detect total and phosphorylated p38 and ERK1/2. IL-1β-treated cells were used as a positive control. To inhibit ERK1/2 activation induced by serotonin and the subsequent effect on SMC marker expression, serum-starved wild-type VSMCs were pretreated with the ERK1/2 inhibitor U0126 (10 µmol/L) (InvivoGen, San Diego, CA, USA; tlrl-u0126), for 30 min before stimulation with or without serotonin for 24 h. Total proteins were then isolated for Western blot analysis to detect expression levels of SM-MHC, SM22α, and SM α-actin. To examine whether AADC expression levels were mediated by the p38 signaling pathway, we pretreated serum-starved wild-type VSMCs for 30 min with 5-MTP or SB203580 (30 µmol/L) (InvivoGen; tlrl-sb20) to inhibit p38 activation prior to stimulation with or without IL-1β for 24 h. Proteins were then prepared for Western blot analysis to detect AADC expression levels.

### Generation of CAG-*hHIOMT373* transgenic mice

After confirming transgene expression in VSMCs, the 4-kb transgenic DNA fragment containing the CAG promoter/enhancer, Flag-*hHIOMT373*, and rabbit globin polyA sequence was isolated, purified, and injected into the pronuclei of fertilized C57BL/6J mouse eggs (National Health Research Institutes (Taiwan), Core Transgenic Mouse Facility). A total of 3 separate injections were performed. Tail lysate containing genomic DNA was prepared from ~ 0.5 cm tail biopsies of potential founders using DirectPCR Lysis Reagent (Tail) (Viagen Biotech, Los Angeles, CA, USA; #102-T) according to the manufacturer’s protocol. Transgenic mice were identified by PCR using the prepared DNA as a template and the primer set (upper primer p1: 5′-TGGATTACAAGGATGACGACG-3′ and lower primer p2: 5′-CTTCAGGGACACACAGATGT-3′) to amplify a 240-bp fragment. The mouse *Csrp2* gene is a diploid gene and has 2 copies [28]. We performed PCR to amplify a 563-bp fragment from the *Csrp2* gene using the upper primer 5′-CCTGGGGCTTAGTGGTTTG-3′ and the lower primer 5′-CCTGAGGAAAGAGTGACTAA-3′. To determine transgene copy number, the ratio of the intensity of the 240-bp transgene fragment to the 563-bp *Csrp2* fragment was calculated and multiplied by 2. Nine founders were identified, and germline transmission was determined by PCR genotyping using tail biopsy DNA.

### Characterization of CAG-*hHIOMT373* transgenic mice

To characterize transgene expression in the different CAG-*hHIOMT* transgenic mouse lines, we extracted proteins from the aorta and different tissues, including the lung, heart, kidney, brain, spleen, and liver for Western blot analysis to detect transgene expression. Proteins from wild-type mouse tissues were used as a negative control. The Y79 cells (Bioresource Collection and Research Center, Hsinchu, Taiwan; #60422) are a human retinoblastoma cell line that expresses the hHIOMT345 isoform, and thus Y79 cell extracts served as a positive control for hHIOMT [6]. The anti-hHIOMT antibody (Abcam; ab180511) recognized Flag-hHIOMT373 as a 43 kDa protein and hHIOMT345 as a 38 kDa protein. The blots were subsequently hybridized with a GAPDH antibody to verify loading.

To detect Flag-hHIOMT and 5-MTP expression, we performed double immunofluorescence staining on the aortic sections of wild-type and different CAG-*hHIOMT373* transgenic mouse lines. Following antigen retrieval and blocking of non-specific binding with 5% BSA, sections were incubated with an anti-Flag antibody (Sigma-Aldrich; F3165) and anti-5-MTP antibody (GenScript; custom-made [24]) at 4 °C overnight and then incubated with a donkey anti-mouse IgG (H&L), Alexa Fluor™ 488 (green fluorescence) secondary antibody (Invitrogen; A-21202) to detect Flag-hHIOMT and goat anti-rabbit IgG H&L (Alexa Fluor^®^ 594, red fluorescence) secondary antibody (Abcam; ab150080) to detect 5-MTP. For immunohistochemical analysis, aortic sections were subjected to antigen retrieval, followed by blocking non-specific binding with 5% BSA. To detect hHIOMT and 5-MTP expression, sections were then incubated with anti-hHIOMT antibody (Abcam; ab180511) and anti-5-MTP antibody (GenScript; custom-made [24]) at 4 °C overnight. DAB (3,3′-Diaminobenzidine) staining was then performed to develop brown color for positive staining with an HRP-conjugated secondary antibody (Dako; K4003), counterstained with Gill’s hematoxylin V (Muto Pure Chemical, Tokyo, Japan; #20,036) to stain nuclei blue-purple color. Slides were then mounted with Surgipath mount (Leica, Wetzlar, Germany; #3801731).

### Generation of SMC-*hHIOMT373* transgenic mice

The conditional transgenic mice overexpressing hHIOMT373 with the emerald green fluorescent protein (emGFP) reporter gene were generated by the genetically modified mouse production service of the National Laboratory Animal Center (Taipei, Taiwan). A bacterial artificial chromosome (BAC) clone that contained the mouse *Actb* (beta-actin) gene was obtained through screening a BAC library by the Center. A synthesized DNA fragment containing a 5′ loxP site, part of the Actb intron 1 sequence, emGFP-polyA-NeoR, and a 3′ loxP site (loxP-Actb intron 1-emGFP-polyA-NeoR-loxP) and a Flag-tagged-hHIOMT373-polyA DNA fragment (excised from the pcDNA3.1 + C-(K)-DYK-*hHIOMT373* expression plasmid) were inserted into the intron 1 and exon 2 of the Actb gene on the BAC DNA, respectively, by Red/ET recombineering technology [29] to create a conditional transgenic construct (Additional file 2: Fig. S1). The modified BAC DNA was then isolated and injected into fertilized eggs of C57BL/6J mice to produce Actb-emGFP-*hHIOMT373*^WT/flox^ conditional transgenic mice (Actb-emGFP), which expressed emGFP throughout the body (custom-made by the National Laboratory Animal Center, Taipei, Taiwan).. The founder mice were genotyped by PCR using the primer set (F1 primer: 5′-CCGAAAGTTGCCTTTTATGGCTC-3′ and B1 primer: 5′-GCTGCAAAGAGTCTACACGCTAGG-3′) to amplify a wild-type 320-bp fragment or a transgenic 423-bp fragment. After confirming germline transmission, we examined whether the hHIOMT373 transgene was leaky in the Actb-emGFP-*hHIOMT373* mice. Total proteins from different tissues were isolated from the conditional transgenic mice to examine emGFP and hHIOMT373 expression. Western blot analysis showed that the Actb-emGFP-*hHIOMT373* mice expressed emGFP but not hHIOMT in tissues, including the aorta, femoral artery, heart, and kidney, while wild-type mice did not express either emGFP or hHIOMT, indicating the hHIOMT373 transgene in the conditional mice was not leaky (Additional file 2: Fig. S2). Actb-emGFP-*hHIOMT373* mice were bred with SM22α-Cre mice in a C57BL/6J background (Jackson Laboratory, Bar Harbor, ME, USA; #017491) to produce double transgenic mice in which the emGFP was removed in the tissues that expressed Cre recombinase, and Flag-hHIOMT373 began to be expressed instead. The green fluorescence of emGFP in the aorta and femoral arteries was observed by exposing them to UV light in the dark environment. The Cre genotype was confirmed by PCR using the primer set (Cre400-F primer: 5′-CGATGCAACGAGTGATGAGGTTC-3′ and Cre400-R primer: 5′-CATGAGTGAACGAACCTGGTCG-3′) to amplify a 400-bp fragment.

### RNA isolation and real-time quantitative RT-PCR (qRT-PCR)

Mouse wild-type and *hHIOMT373* transgenic VSMCs were quiesced for 48 h before total RNA isolation for qRT-PCR analysis. Human aortic smooth muscle cells (HASMCs; Applied Biological Materials, Richmond, BC, Canada; T0515) were quiesced for 3 d before treatment with or without human IL-1β (10 ng/mL; PeproTech, #200-01B) for 3 d. The CM from HASMCs was collected for 5-MTP measurement. Total RNA was then extracted from cells using RNAzol^®^ RT (Molecular Research Center, Cincinnati, OH, USA; RN 190) according to the manufacturer’s instructions. Two micrograms of total RNA from each sample were reverse transcribed into cDNA using SuperScript™ III Reverse Transcriptase (Invitrogen, #18080051). qRT-PCR was performed using the QuantStudio™ 3 Real-Time PCR System (Applied Biosystems, Waltham, MA, USA) and SYBR™ Green PCR Master Mix (Thermo Fisher, Waltham, MA, USA; #4367659). A specific primer set was used to amplify *mHiomt* (upper primer: 5′-TCTACCTGGCGGGCACC-3′ and lower primer: 5′- CGACCTGTAGATGGCGGTG-3′), while mouse *Gapdh* was used as an internal control for normalization (upper primer: 5′- CATCACTGCCACCCAGAAGACTG-3′ and lower primer: 5′-ATGCCAGTGAGCTTCCCGTTCAG-3′). The primer set (upper primer: 5′- GTCAGCCCGACGTCACAAT-3′ and lower primer: 5′- GCCAAACGTCTCCAGGTACTG-3′) was used to amplify *hHIOMT* and human *GAPDH* (upper primer: 5′-GATGACATCAAGAAGGTGGTGA-3′ and lower primer: 5′- GTCTACATGGCAACTGTGAGGA-3′) was used as an internal control for normalization. Each reaction was carried out in triplicate. For mouse VSMC samples, the endogenous *mHiomt* and exogenous *hHIOMT* expression were quantified using the ΔCt (delta threshold cycle) method, normalizing *mHiomt* and *hHIOMT* expression to mouse *Gapdh*. For HASMC samples, the ΔΔCt (comparative threshold cycle) method was used to determine relative expression levels of *hHIOMT* by normalizing to human *GAPDH* and was expressed relative to the vehicle control group.

### 5-MTP measurement by UPLC-mass spectrometry

To measure mouse circulating 5-MTP levels, blood was drawn from wild-type and *hHIOMT373* transgenic mice, and plasma was prepared to assess 5-MTP levels. Alternatively, CM was prepared from HASMCs for 5-MTP measurement. The amount of 5-MTP was quantified using an ultraperformance liquid chromatography (UPLC) system coupled to a G6495C triple quadrupole mass spectrometer (Agilent Technologies, Santa Clara, CA, USA), as previously described [6, 18]. Briefly, chromatographic separation was performed using an Acquity UPLC system (Waters) equipped with a BEH C18 column (1.7 µm, 2.1 × 100 mm). The mobile phase consisted of solvent A (water with 0.1% formic acid, v/v) and solvent B (acetonitrile with 0.1% formic acid, v/v). A linear gradient elution was applied as follows: 0–0.5 min: 5% B, 0.5–4.0 min: gradient from 5 to 95% B, 4.0–5.5 min: 95% B, 5.5–5.6 min: gradient return to 5% B, and 5.6–9.0 min: re-equilibration at 5% B. The column temperature was maintained at 40 °C, and the injection volume was 2 μL. Mass spectrometric detection was conducted using the G6495C triple quadrupole system with an electrospray ionization source operated in both positive and negative ion modes. The capillary voltages were set at + 3000 V and − 2500 V for the respective modes. Data acquisition was performed in multiple reaction monitoring mode. Quantification of 5-MTP was performed using Agilent MassHunter software. Calibration curves were generated using pure 5-MTP standards at concentrations ranging from 0.0 to 25 nmol/L.

### Mouse guidewire-induced femoral artery injury

All experimental procedures were performed in accordance with NIH guidelines for the care and use of laboratory animals and approved by the Institutional Animal Care and Use Committee of National Health Research Institutes, Taiwan (NHRI-IACUC-107056-A and NHRI-IACUC-110051-A). Approximately 10 weeks old (20–25*g*) male wild-type C57BL/6J mice or *hHIOMT373* transgenic mice were subjected to a neointima formation model of femoral artery denudation injury [30]. Mice were anesthetized with isoflurane vapor by inhalation, 4–5% initially and 1–3% during the procedure, to achieve appropriate sedation. Endoluminal injury to the left common femoral artery was then performed to denude the endothelium with 5 passages of a 0.014″-diameter guidewire (Hi-Torque Cross-it 100XT, with hydrocoat hydrophilic coating; Abbott) and kept the wire in the vessel for 1 min after the 5th passage before pulling it out.

### Histological analysis and immunohistochemistry

Wild-type and *hHIOMT373* transgenic mice were anesthetized with an overdose (500–750 mg/kg) of tribromoethanol solution (avertin) by IP injection. Avertin is approved by NHRI IACUC for short-term (up to 1 h) surgical procedures in mice with a single injection. Avertin (12.5 mg/mL) was prepared by dissolving 0.5*g* of 2,2,2-tribromoethanol (Sigma-Aldrich; T84802) in 1 mL of tertiary amyl alcohol (Sigma-Aldrich; A1685) by heating to 37 °C; distilled water was then added to a final volume of 40 mL and filtered to sterilize. Within 10–15 min of the avertin injection, after assessing adequate depth of anesthesia via lack of response to toe pinch, the skin and muscles were cut through, and then the diaphragm, to expose the heart. The mouse was then perfused with PBS through the heart, followed by 10% neutral-buffered formalin (Sigma-Aldrich; HT501128), and the aortas and other tissues were harvested and further fixed in 10% formalin at 4 °C overnight before processing and embedding in paraffin. Serial 4-μm cross sections were collected for immunostaining with an hHIOMT antibody to detect hHIOMT transgene expression. At indicated time points following femoral artery injury, mice were anesthetized as above, and uninjured contralateral and injured left femoral arteries were then carefully dissected, excised, and further fixed in 10% formalin at 4 °C overnight before processing and embedding in paraffin. Serial 4-μm longitudinal sections of the femoral arteries were collected. Sections were stained with H&E for morphology and Verhoeff's stain (Sigma-Aldrich; HT25A) for delineation of elastin layers, respectively. Three sets of sections at 60-µm intervals were used for morphometry; the intimal and medial areas were measured using NIH ImageJ 1.53i software, and the intima-to-media ratio was calculated essentially as described [22]. Arterial sections were immunostained with antibodies for hHIOMT, 5-MTP, matrix metalloproteinase 2 (MMP2) (Abcam; ab37150), or AADC. For analysis of hHIOMT and 5-MTP expression in the tunica media of human vascular tissues, frozen sections of normal and arteriosclerotic human arteries were obtained from BioChain Institute (Newark, CA, USA; T1234013 and T1236013Hd-4, respectively). Sections were processed and immunostained for hHIOMT and 5-MTP. Hematoxylin was used for nuclear counterstaining. For immunohistochemistry, the antibodies used are described in Additional file 1.

### Statistical analyses

Averaged values are presented as mean ± SEM. Student’s *t*-test (two-tailed, unpaired) was performed to compare the intima/media ratio between wild-type and transgenic mice. Two-tailed, paired Student’s *t*-test was performed to compare the CM 5-MTP levels between control and IL-1β-treated HASMCs. Group comparisons were conducted using one-way ANOVA followed by Tukey's test. Statistical significance was recognized at *P* < 0.05.

## Results

### *hHIOMT373* overexpression attenuates proinflammatory cytokine-induced p38 MAPK activation in VSMCs

**Fig. 1:**
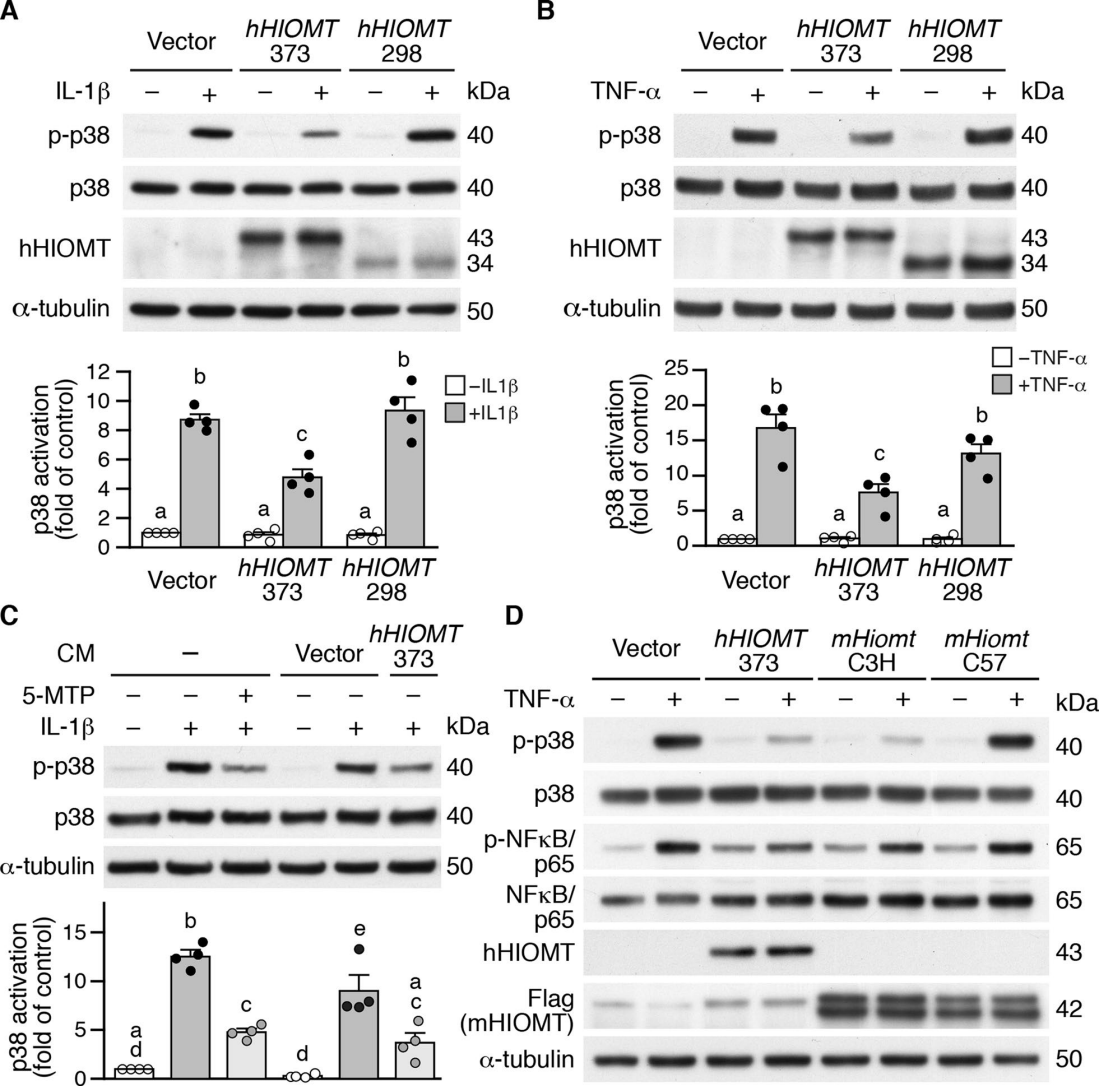
HIOMT expression attenuates proinflammatory cytokine-induced p38 MAPK activation in VSMCs. **A-B** Wild-type VSMCs were electroporated with a control vector or *hHIOMT* expression plasmids *hHIOMT373* and *hHIOMT298* for 24 h, stimulated with or without **A** IL-1β or **B** TNF-α for 15 min, and proteins were prepared for Western blotting to detect hHIOMT expression, p38 MAPK activation, total p38, and α-tubulin. A representative blot is shown (n = 4). Quantitation of p38 activation (different letters indicate significant differences between groups as determined by one-way ANOVA followed by Tukey's test). **C** CM from vector- and *hHIOMT373*-transfected wild-type cells was collected 40 h after transfection and used to incubate VSMCs for 24 h before stimulation with IL-1β for 15 min, and total proteins were prepared. As a control, cells without any transfection were pretreated with or without 5-MTP and then stimulated with or without IL-1β for 15 min, and total proteins were prepared. Western blot analysis was performed to detect total and phosphorylated p38 and α-tubulin. A representative blot is shown (n = 4). Quantitation of p38 activation (different letters indicate significant differences between groups as determined by one-way ANOVA followed by Tukey's test). **D** Wild-type VSMCs were transfected with vector or *hHIOMT373* expression plasmid or mouse *Hiomt* (*mHiomt*) expression plasmids *mHiomt*-C3H (from C3H strain) and *mHiomt*-C57 (from C57BL/6J strain) for 24 h, then stimulated with or without TNF-α for 15 min. Proteins were prepared for Western blotting to detect total and phosphorylated p38, NFκB/p65, hHIOMT, mHIOMT (using Flag antibody), and α-tubulin

To investigate the potential role of *HIOMT* in VSMCs and because hHIOMT345 is mainly expressed in pineal cells [2], we examined the function of hHIOMT373 and hHIOMT298 in VSMCs. Mouse primary VSMCs were transfected with a control vector or Flag-tagged *hHIOMT* expression plasmid and then stimulated with IL-1β or TNF-α. Previous studies have established that 5-MTP suppresses inflammatory mediator-mediated p38 MAPK activation [3, 17, 18]. We reasoned that if HIOMT is the synthetic enzyme of 5-MTP, overexpression of HIOMT should generate 5-MTP and exert similar effects as exogenously administered 5-MTP. As such, suppression of p38 MAPK and/or NFκB activation was used as an indicator to assess HIOMT enzymatic activity. IL-1β increased p38 phosphorylation in vector-transfected cells. In comparison, overexpression of hHIOMT373 decreased ~ 50% of p38 phosphorylation, whereas hHIOMT298 did not significantly affect p38 activation (Fig. 1A). Western analysis using the hHIOMT antibody (which did not recognize mouse HIOMT (mHIOMT)) confirmed the expression of the two isoforms. As with IL-1β, TNF-α stimulated p38 activation, which was attenuated by hHIOMT373 but not by hHIOMT298 expression (Fig. 1B). These results indicate that in VSMCs, hHIOMT373 rather than hHIOMT298 is responsible for suppressing inflammatory mediator-elicited p38 activation. Earlier studies have shown that 5-MTP is released into the extracellular milieu for anti-inflammatory actions [18]. We thus collected CM from vector- or hHIOMT373-transfected VSMCs and tested whether CM had a similar effect as 5-MTP. Consistent with the previous report [22], 5-MTP decreased ~ 50% of IL-1β-induced p38 phosphorylation in VSMCs (Fig. 1C). Compared with vector-transfected CM, *hHIOMT373*-transfected CM reduced IL-1β-induced p38 phosphorylation to ~ 45% (Fig. 1C), indicating *hHIOMT373*-transfected CM had a similar effect as exogenously added 5-MTP on attenuating p38 activation. These data support that hHIOMT373 is likely the active isoform and responsible for generating 5-MTP in VSMCs.

The C57BL/6J mouse strain has been routinely used in many animal models. Because of two mutations in the mouse *Hiomt* gene, *mHiomt*-C57 has been suggested to have an enzymatic defect and impaired production of 5-MTP [25]. We therefore transfected *mHiomt*-C57 (with 2 mutations), *mHiomt*-C3H (from the C3H strain, without mutations), or *hHIOMT373* expression plasmids into VSMCs and treated cells with or without TNF-α. Expression of exogenous HIOMT was confirmed by either hHIOMT antibody (for human isoform) or Flag antibody (for mouse HIOMT) (Fig. 1D). hHIOMT373 and mHIOMT-C3H repressed TNF-α-induced p38 phosphorylation. Intriguingly, mHIOMT-C57 did not repress p38 phosphorylation. We also observed a similar effect on NFκB-p65 activation (Fig. 1D). These data suggest that C57 strain VSMCs have low baseline HIOMT activity and that hHIOMT373 and mHIOMT-C3H can functionally substitute 5-MTP in VSMCs.

### hHIOMT and 5-MTP are induced by IL-1β in HASMCs and are increased in the tunica media of arteriosclerotic human arteries

To investigate whether human VSMCs express *hHIOMT*, we treated HASMCs with or without IL-1β. qRT-PCR analysis revealed that IL-1β increased *hHIOMT* gene expression levels by ~ two fold (Additional file 2: Fig. S3A). In line with increased *hHIOMT* expression, IL-1β enhanced 5-MTP levels in the CM of HASMCs (Additional file 2: Fig. S3B). Furthermore, immunohistochemical analysis of human vascular tissues showed that normal arteries expressed very low levels of hHIOMT and 5-MTP in the tunica media, whereas their levels were increased in the media of the arteriosclerotic arteries (Additional file 2: Fig. S3C). These results suggest that the induction of *hHIOMT* and 5-MTP by inflammatory cytokines or under pathological conditions conceivably serves as an endogenous protective mechanism, similar to inducible stress response genes, such as heme oxygenase-1 [31].

### Generation and characterization of CAG-*hHIOMT373* transgenic mice

**Fig. 2:**
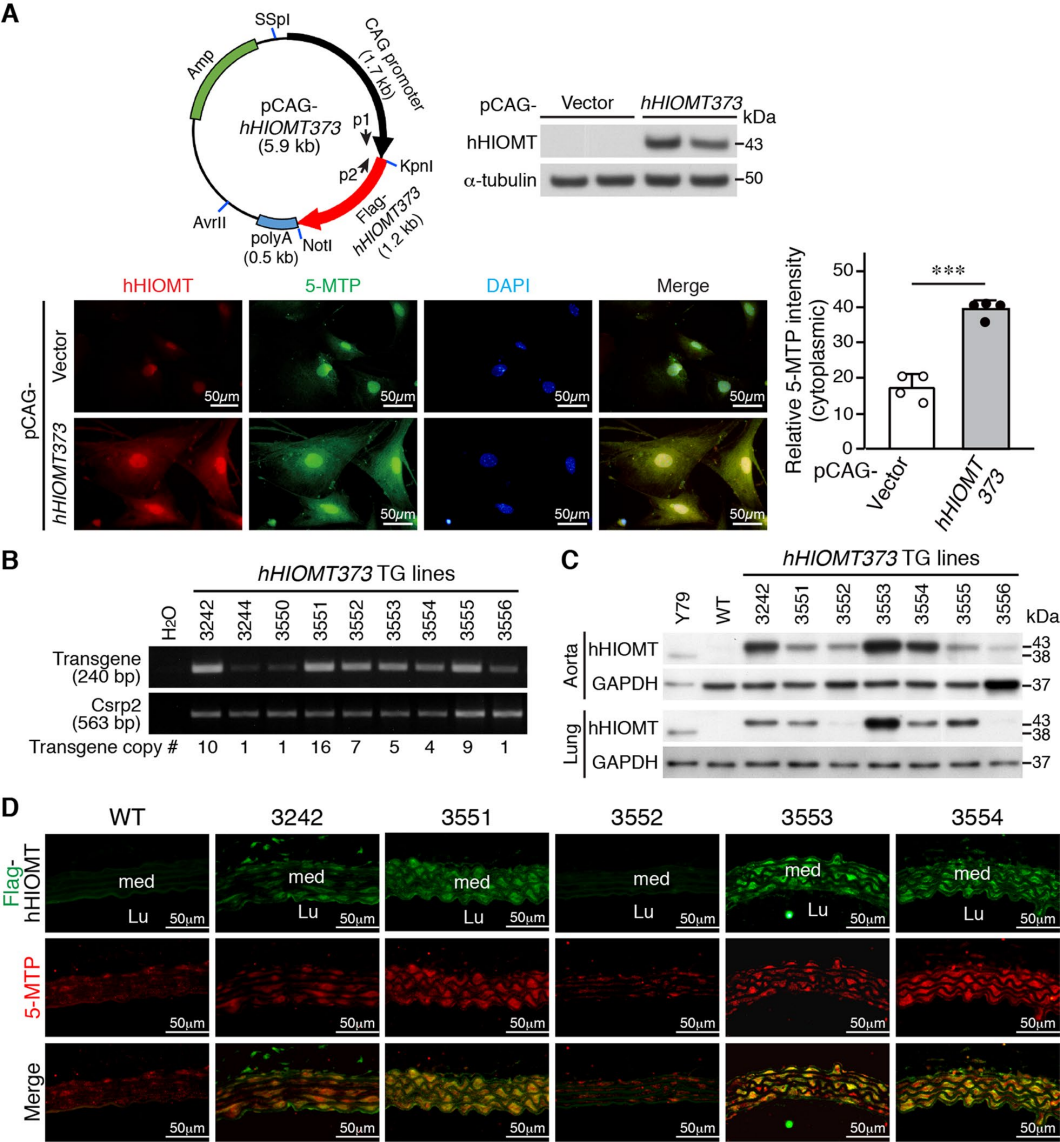
Generation and characterization of CAG-*hHIOMT373* transgenic mice. **A** Flag-tagged *hHIOMT373* cDNA was cloned into a pCAG vector under the control of a CAG promoter. PCR primers (p1 and p2) for genotyping are indicated. The pCAG vector or pCAG-*hHIOMT373* transgenic construct was transfected into primary wild-type VSMCs in duplicate. Proteins were prepared for Western blotting 24 h after transfection to detect Flag-hHIOMT373 (43 kDa) or α-tubulin for loading control. Alternatively, transfected VSMCs were fixed, and immunostaining was performed with antibodies for hHIOMT (red) and 5-MTP (green); nuclei were stained with DAPI (blue). Colocalization of HIOMT and 5-MTP is revealed by the yellow color in merged images. Scale bar, 50 µm. The cytoplasmic green fluorescence of 5-MTP staining was quantified and expressed as the mean intensity of 5-MTP of the positive cell relative to that of the negative cell. ***P < 0.001 (n = 4; unpaired Student’s *t*-test). **B** Tail DNA from a total of 9 potential founders was isolated and subjected to genotyping by PCR. H_2_O or the transgenic plasmid was used for negative and positive control, respectively. A 240-bp fragment indicates the presence of a transgene. Transgene copy number was determined using the mouse *Csrp2* gene as a reference. **C** Total proteins were isolated from aorta and lungs from wild-type (WT) and transgenic lines for Western blot analysis. WT served as a negative control. The human Y79 cell line expresses the *hHIOMT345* isoform, encoding a 38 kDa protein, and thus Y79 cell extracts served as a positive control for hHIOMT. Blots were hybridized to hHIOMT antibody and GAPDH for loading control. **D** Immunofluorescence staining of WT and 5 lines of hHIOMT373-transgenic aortas with Flag (green) and 5-MTP (red) antibodies. The yellow color in the merged images indicates overlapping expression of hHIOMT373 and 5-MTP. Lu, lumen; med, media. Scale bar, 50 µm

To investigate whether *hHIOMT373* can protect against inflammatory vascular diseases in animals, we first generated a transgenic construct, pCAG-*hHIOMT373* expressing *hHIOMT373* cDNA under the control of a strong synthetic promoter, CAG (Fig. 2A). Transfection of the transgenic construct into VSMCs showed overexpression of the hHIOMT373 transgene by Western analysis (Fig. 2A). Immunofluorescence staining revealed a low-level, non-specific background staining of vector-transfected cells, while transfection of pCAG-*hHIOMT373* resulted in strong staining of hHIOMT373 (red) in the cytoplasmic and nuclear regions (Fig. 2A). Importantly, immunostaining of 5-MTP (green) showed a similar pattern of expression. Colocalization of hHIOMT373 and 5-MTP was revealed by yellow color in merged images (Fig. 2A), indicating that hHIOMT373 expression was correlated with 5-MTP production. Quantitation of the green fluorescence intensity of 5-MTP revealed that cells transfected with *hHIOMT373* displayed significantly higher 5-MTP levels than those of the vector (Fig. 2A). Based on this information, we then used the pCAG-*hHIOMT373* construct to generate transgenic mice for in vivo experiments.

Genotyping revealed that a total of 9 transgenic founders (in the C57BL/6J strain) were obtained (Fig. 2B). Transgene copy number was determined by using the mouse *Csrp2* gene as an internal reference and plasmid DNA as a standard in semi-quantitative PCR reactions. The transgene copy number ranged from 1 copy to 16 copies (Fig. 2B). Breeding of founders with wild-type mice indicated 8 founders (except 3244) had germline transmission to offspring. Western blotting showed that many lines had transgene expression in the aorta, lung, and other tissues (Fig. 2C and Additional file 2: Fig. S4). Immunofluorescence staining of aortic sections using Flag antibody to detect hHIOMT373 transgene (green) revealed that line 3552 eventually lost transgene expression in the aorta while lines 3242, 3551, 3553, and 3554 maintained hHIOMT373 expression (Fig. 2D). Importantly, 5-MTP expression (red) followed the same pattern as Flag-hHIOMT373, and the merged images confirmed colocalization of the transgene and 5-MTP (Fig. 2D). Immunohistochemistry further confirmed the expression of the hHIOMT373 transgene and 5-MTP in the aortic medial VSMC layer from lines 3242, 3551, 3553, and 3554 (Additional file 2: Fig. S5).

### Overexpression of *hHIOMT373* protects against intimal hyperplasia following arterial denudation injury in mice

**Fig. 3:**
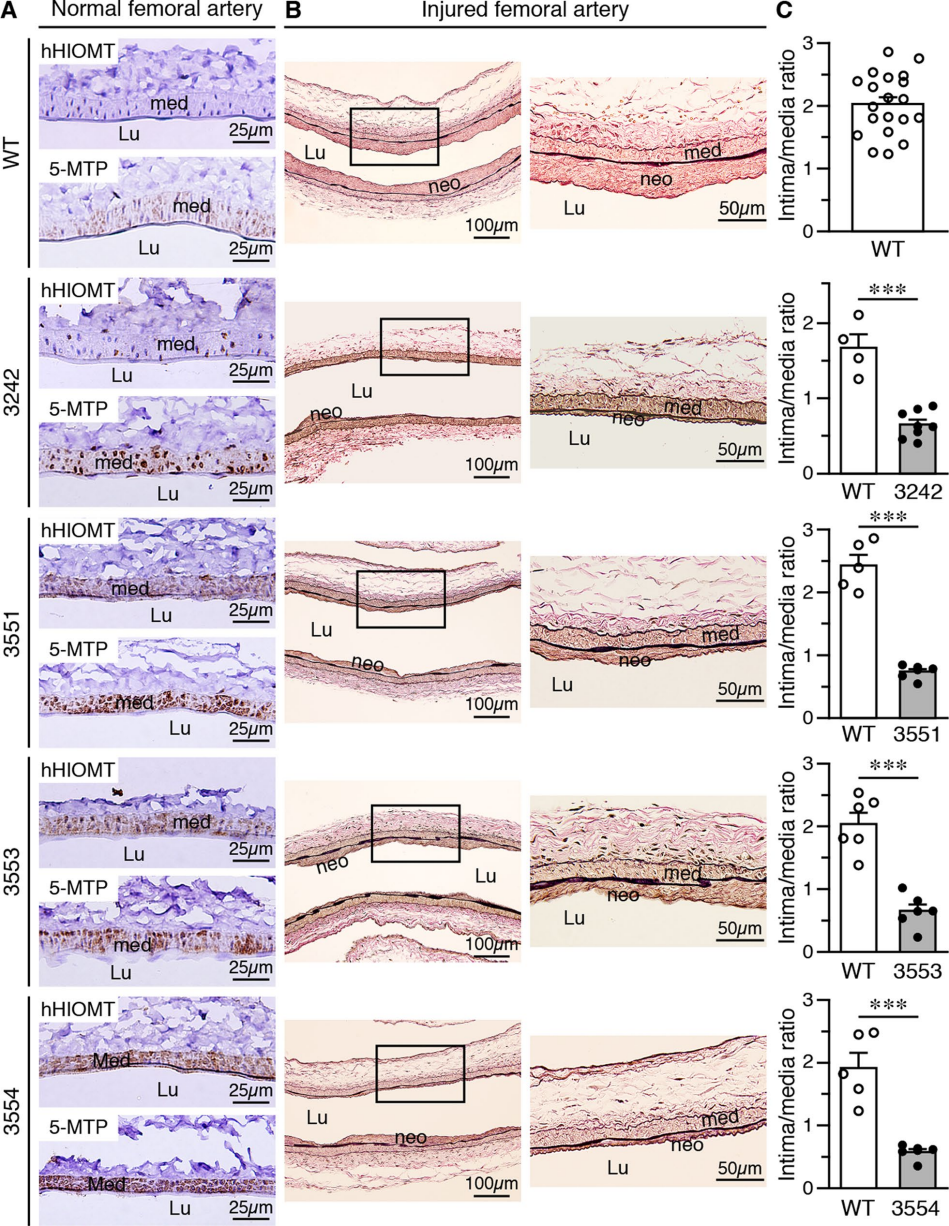
Overexpression of *hHIOMT373* protects against intimal hyperplasia following arterial denudation injury. **A** Immunostaining of femoral arteries from wild-type (WT) and transgenic (TG) mice with hHIOMT (top) and 5-MTP (bottom) antibodies. Scale bar, 25 µm. **B** Each transgenic line and corresponding WT mice (TG3242 n = 8, WT n = 4; TG3551 n = 6, WT n = 5; TG3553 n = 6, WT n = 6; TG3554 n = 6, WT n = 5) were subjected to femoral artery denudation injury, and vessels were harvested 4 weeks later. Arterial sections (left column) were stained with Verhoeff’s stain to delineate elastin (scale bar, 100 µm). Higher magnification of the boxed area is shown at the respective right panel (scale bar, 50 µm). **C** The areas of neointima and media were quantified and expressed as an intima/media ratio. The intima/media ratio of all WT mice (top panel, n = 20) was calculated from pooled control WT for different TG lines. Med, media; Lu, lumen; neo, neointima. ***P < 0.001 (two-tailed, unpaired Student’s *t*-test)

We next wanted to test whether overexpression of *hHIOMT373* protected against vascular injury in mice. *hHIOMT373* transgene expression was first evaluated in mouse femoral arteries. Immunostaining showed that the hHIOMT373 transgene was not detected in wild-type femoral arteries; in comparison, the hHIOMT373 transgene was expressed in the medial layer of femoral arteries of lines 3242, 3551, 3553, and 3554 (Fig. 3A). Consistent with hHIOMT immunostaining and compared with wild-type, transgenic mice had increased 5-MTP levels (Fig. 3A). Wild-type and transgenic mice were then subjected to femoral artery denudation injury, vessels harvested, and neointima formation examined after 4 weeks. Compared with the large neointima in wild-type mice, transgenic mice had smaller neointimas in all the lines examined (Fig. 3B). Quantitative analysis revealed a significantly reduced intima/media ratio in mice overexpressing *hHIOMT373* (Fig. 3C), indicating that *hHIOMT373* protects against injury-induced vascular remodeling. Because hHIOMT373 expression was correlated with 5-MTP production (Fig. 2A) and 5-MTP can be secreted into the extracellular milieu [18], we next examined whether the circulating 5-MTP levels were increased in the *hHIOMT373* transgenic mice. Indeed, hHIOMT373 expression increased plasma concentrations of 5-MTP to ~ 2.7 nmol/L from ~ 1.9 nmol/L of wild-type mice (Additional file 2: Fig. S6A). qRT-PCR analysis using RNA from mouse VSMCs further demonstrated that both wild-type and *hHIOMT373* transgenic VSMCs expressed comparable levels of endogenous *mHIOMT* (Additional file 2: Fig. S6B, left panel). Furthermore, transgenic VSMCs expressed elevated levels of the exogenous *hHIOMT373* transgene, whereas wild-type cells had no expression of the exogenous transgene (Additional file 2: Fig. S6B, right panel).

### Vascular smooth muscle cell-specific expression of *hHIOMT373* attenuates intimal hyperplasia following arterial injury

**Fig. 4:**
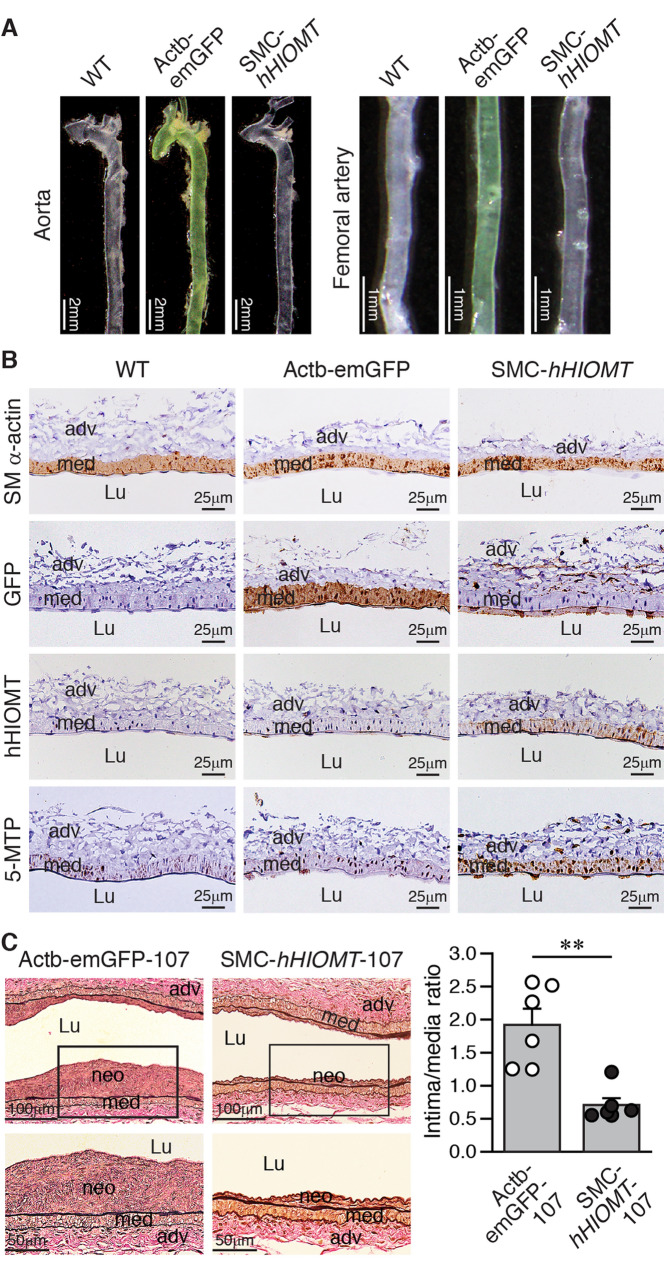
Vascular smooth muscle-specific expression of hHIOMT attenuates neointima formation after injury in mice. **A** Green fluorescence of emGFP in the aorta (scale bar, 2 mm) and femoral artery (scale bar, 1 mm) of wild-type (WT), Actb-emGFP-*hHIOMT373* (Actb-emGFP), and SMC-*hHIOMT373* (SMC-*hHIOMT*) mice is shown. **B** Histological analysis of SM α-actin, GFP, hHIOMT, and 5-MTP expressions by immunostaining (brown) in femoral arteries. Scale bar, 25 µm. **C** Actb-emGFP line 107 (n = 6) and SMC-*hHIOMT* line 107 mice (n = 6) were subjected to femoral artery denudation injury. Vessels were harvested 4 weeks after injury for histological analysis. Verhoeff’s elastin stain was performed on femoral artery longitudinal sections (top row). Scale bar, 100 µm. Representative images are shown. Higher magnification of the boxed area is shown at the respective bottom panel (scale bar, 50 µm). The intima-to-media ratio was calculated. **P < 0.01 (two-tailed, unpaired Student’s *t*-test). Lu, lumen; neo, neointima; adv, adventitia; med, media

To confirm that the vascular protection is due specifically to the expression of *hHIOMT373* in VSMCs but not secondary effects of *hHIOMT373* expression from other tissues, we generated VSMC-specific expression of *hHIOMT373* (SMC-*hHIOMT*) transgenic mice, which expressed hHIOMT373 instead of emGFP in VSMCs; thus, the aorta and femoral arteries were no longer green (Fig. 4A). SM α-actin-positive immunostaining (brown) marked the medial smooth muscle layer of the arteries (Fig. 4B). GFP staining showed that emGFP was expressed in the medial layer of Actb-emGFP but not in wild-type or SMC-*hHIOMT* transgenic mouse arteries (Fig. 4B). Compared to wild-type or Actb-emGFP mice, the expression levels of transgene hHIOMT and 5-MTP, the enzymatic product of HIOMT, were higher in SMC-*hHIOMT* mouse arteries (Fig. 4B). Interestingly, a closer examination showed that although the endothelium of SMC-*hHIOMT* arteries did not express hHIOMT but had more 5-MTP-positive endothelial cells compared with wild-type or Actb-emGFP arteries (Additional file 2: Fig. S7), suggesting that 5-MTP produced in the medial SMCs can be secreted into the extracellular space and enter endothelial cells. The crosstalk between SMCs and ECs certainly warrants further investigation. Four weeks following wire injury, Verhoeff’s elastin staining revealed that large neointima was present in the injured arteries from Actb-emGFP mice, whereas neointima was smaller and had a reduced intima/media ratio in the injured arteries from both lines (107 and 110) of SMC-*hHIOMT* mice (Fig. 4C and Additional file 2: Fig. S8). These results indicate that SMC-specific expression of *hHIOMT373* protects against intimal hyperplasia after arterial injury.

### *hHIOMT373* expression reduces arterial MMP2 levels after injury and decreases inflammatory cytokine-induced VSMC inflammation, proliferation, and migration

We next began to investigate the vascular protective mechanisms of *hHIOMT373*. As MMP2 plays an important role in vascular remodeling [32], we examined MMP2 levels in control and 2-week injured femoral arteries. Injury increased MMP2 levels in the arteries, including neointima, media, and adventitia in wild-type mice; in comparison, injured transgenic arteries had smaller neointima and lower levels of MMP2 (Additional file 2: Fig. S9A). We treated primary cultured VSMCs from wild-type and *hHIOMT373* mice with IL-1β. Western analysis revealed that IL-1β-increased MMP2 level was attenuated in transgenic cells (Additional file 2: Fig. S9B). These results indicate that, as with 5-MTP [23], *hHIOMT373* overexpression protects against vascular remodeling and is at least in part through mitigating MMP2 levels. Further analysis showed that IL-1β-induced activation of p38 and p65 was reduced in transgenic VSMCs (Additional file 2: Fig. S9C), indicating that transgenic cells possessed anti-inflammatory properties. Indeed, transgenic cells had attenuated proliferative responses to IL-1β and TNF-α (Additional file 2: Fig. S9D). Interestingly, similar to a previous finding that 5-MTP did not affect growth factor PDGF-induced VSMC proliferation [22], *hHIOMT373* expression did not affect PDGF-stimulated cellular proliferation (Additional file 2: Fig. S9D). On the other hand, transgenic cells exhibited significantly reduced migratory capacity upon PDGF, IL-1β, and TNF-α stimulation in wound-closure assays (Additional file 2: Fig. S9E).

### Treatment of 5-MTP or *hHIOMT373* overexpression reduces baseline and IL-1β-induced serotonin production but does not influence the effect of serotonin on VSMC proliferation

**Fig. 5:**
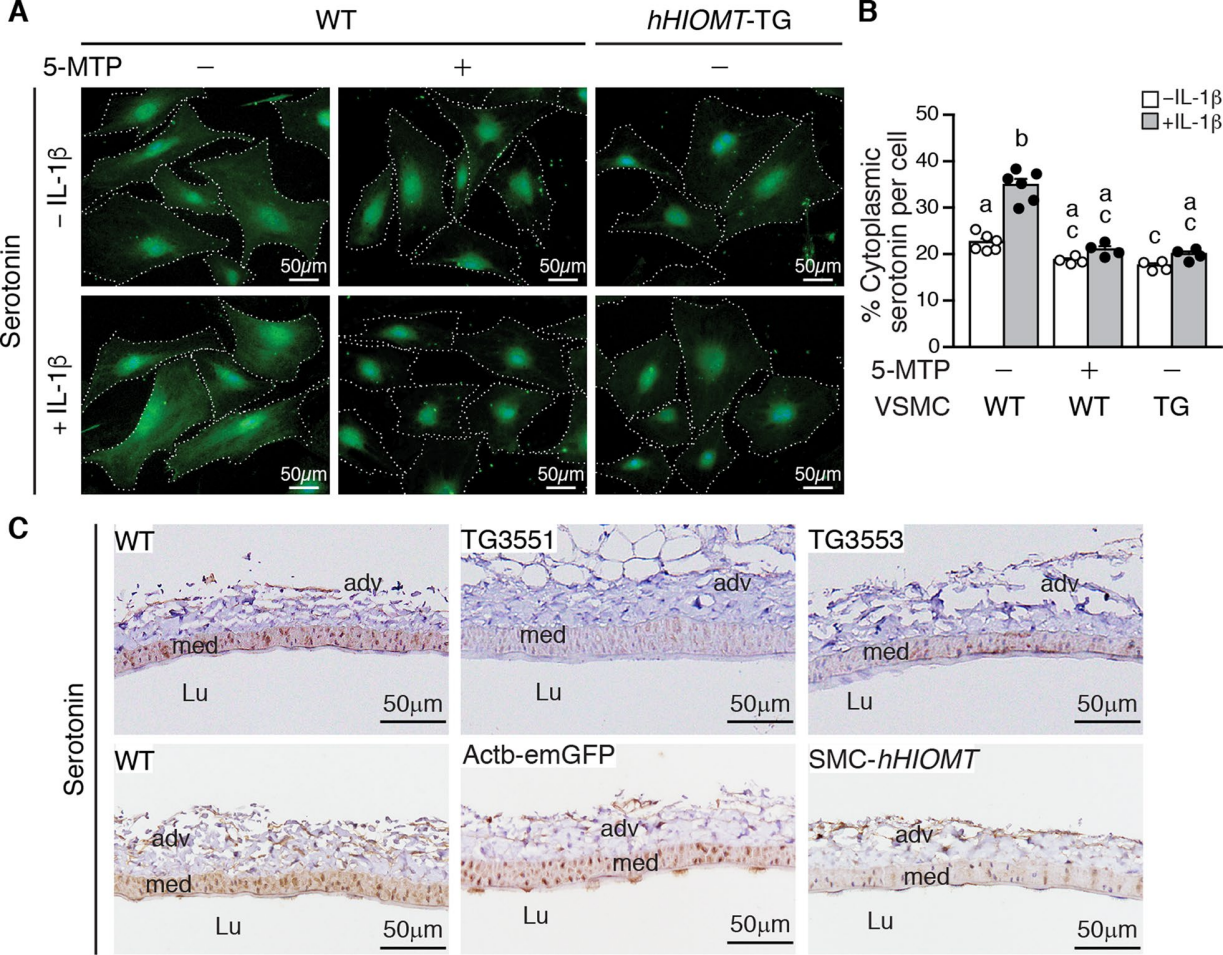
Treatment of 5-MTP or *hHIOMT373* overexpression reduces basal and IL-1β-induced serotonin in VSMCs. **A** Primary cultured wild-type (WT) and *hHIOMT* transgenic (TG) VSMCs were serum-starved, and WT cells were pretreated with or without 100 µmol/L 5-MTP for 24 h. Both WT and TG VSMCs were then treated with or without IL-1β (10 ng/mL) (WT –5MTP –IL-1β, n = 6; WT –5MTP + IL-1β, n = 6; WT + 5MTP –IL-1β, n = 4; WT + 5MTP + IL-1β, n = 4; TG –IL-1β, n = 4; TG + IL-1β, n = 4). Twenty-four hours later, cells were then fixed for immunofluorescence staining with serotonin antibody (green). Each cell is demarcated with a dotted white line. Representative images are shown. Scale bar, 50 µm. **B** Serotonin expression (green fluorescence) from each experiment was measured using ImageJ software from 3 fields (200 × , 5 cells/field) of each condition and expressed as % cytoplasmic positive staining per cell. WT –5MTP –IL-1β, n = 6; WT –5MTP + IL-1β, n = 6; WT + 5MTP –IL-1β, n = 4; WT + 5MTP + IL-1β, n = 4; TG –IL-1β, n = 4; TG + IL-1β, n = 4. Different letters indicate significant differences between groups as determined by one-way ANOVA followed by Tukey's test. **C** Serotonin immunostaining (brown) of mouse femoral arterial sections from WT (n = 8), TG3551 (n = 4), TG3553 (n = 4), Actb-emGFP (n = 2), and SMC-*hHIOMT* (n = 3) transgenic mice. Representative sections are shown. Scale bar, 50 µm. Lu, lumen; adv, adventitia; med, media

Syntheses of serotonin and 5-MTP share the same intermediate, 5-hydroxytryptophan, through two different enzymes, AADC and HIOMT, by the two branches of the serotonin pathway, respectively [4]. Serotonin has been implicated in cardiovascular diseases [33, 34]. We thus investigated whether *hHIOMT373* overexpression or 5-MTP treatment in VSMCs alters tryptophan metabolism under normal physiological and pathophysiological conditions. We first treated wild-type VSMCs with IL-1β in the absence or presence of 5-MTP for 24 h and examined serotonin expression. Immunofluorescence staining revealed a baseline expression of serotonin in the cytosol of VSMCs, and IL-1β increased cytosolic serotonin expression (Fig. 5A). Of note, the nuclear staining appeared to be nonspecific. Treatment with 5-MTP decreased baseline and IL-1β-induced serotonin levels (Fig. 5A). To determine whether overexpression of *hHIOMT373* in VSMCs exerts a similar effect, *hHIOMT373* transgenic VSMCs were treated without or with IL-1β for 24 h, and then immunostaining was performed. The results showed that similar to that of 5-MTP-treated VSMCs, *hHIOMT373* transgenic cells had reduced baseline serotonin levels and attenuated IL-1β-induced serotonin expression (Fig. 5A). Quantitation of cytoplasmic serotonin levels confirmed the findings (Fig. 5B). These results indicate that in VSMCs there is a basal level expression of serotonin, which is inducible by IL-1β, whereas 5-MTP and hHIOMT373 exert similar effects in reducing serotonin levels.

**Fig. 6:**
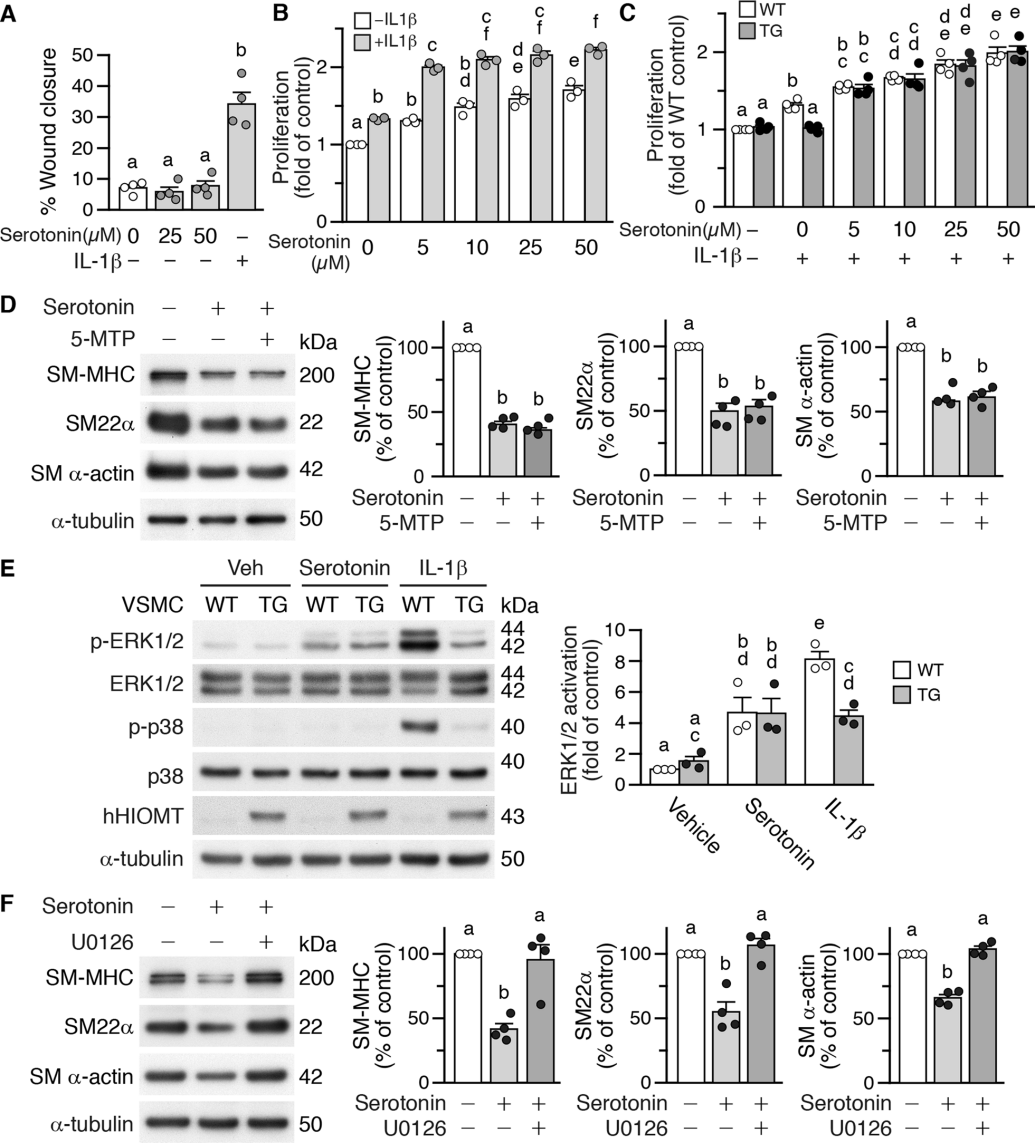
Serotonin reduces VSMC proliferation and marker expression via the ERK1/2 pathway. **A** Serum-starved wild-type (WT) VSMCs were plated, wounded, and then treated with vehicle, different doses of serotonin, or IL-1β (10 ng/mL, as positive control) in starvation medium for 6 h. Wound images were captured at time 0 and at 6 h. Wound closure was quantified, and % wound closure was calculated (n = 4 each). **B** WT VSMCs were stimulated with different doses of serotonin for 24 h in the presence or absence of IL-1β (2 ng/mL), and proliferation was assessed by CCK-8 assays. n = 3 each. **C** Serum-starved WT and transgenic (TG) VSMCs were stimulated with vehicle, IL-1β (2 ng/mL), or 25 µmol/L serotonin + IL-1β (2 ng/mL) for 24 h, and proliferation was assessed. n = 4 each. **D** WT VSMCs were treated with vehicle, serotonin, or serotonin in the presence of 5-MTP (100 µmol/L). Proteins were prepared 24 h later for Western blotting to detect SM-MHC, SM22α, SM α-actin, and α-tubulin as a loading control. Quantitative analysis of VSMC markers, n = 4 each group. **E** WT and TG VSMCs were stimulated with vehicle, serotonin, or IL-1β (10 ng/mL) for 15 min, and proteins were prepared for Western blotting to detect phosphorylated and total ERK1/2 and p38, hHIOMT, and α-tubulin as a loading control. Quantitative analysis of ERK1/2 activation in WT and TG VSMCs. n = 3 for each group. **F** WT VSMCs were treated with vehicle or serotonin in the absence or presence of U0126. Proteins were prepared 24 h later for Western blotting to detect VSMC markers and α-tubulin as a loading control. Quantitative analysis of VSMC markers, n = 4 for each group. Different letters indicate significant differences between groups as determined by one-way ANOVA followed by Tukey's test

We next examined whether *hHIOMT373* suppressed serotonin levels in vivo. Immunostaining revealed that serotonin was expressed in the arterial medial layer of femoral arteries, whereas overexpression of *hHIOMT373* in the TG3551 or TG3553 lines decreased arterial serotonin levels (Fig. 5C, top row). Further confirming this finding, wild-type and Actb-emGFP mouse arteries had similar levels of serotonin expression, whereas SMC-*hHIOMT* transgenic mice had reduced serotonin levels (Fig. 5C, bottom row). These results suggest that HIOMT-derived 5-MTP suppresses VSMC serotonin production, which may be a result of downregulation of its synthetic enzyme, AADC. Given that IL-1β increased VSMC proliferation and migration [22] and that IL-1β increased VSMC serotonin expression, we next examined whether serotonin affected VSMC proliferation and migration. Interestingly, unlike IL-1β, serotonin did not affect VSMC migration (Fig. 6A). In contrast, serotonin dose-dependently increased VSMC proliferation (Fig. 6B, white bars), and IL-1β (2 ng/mL) further increased cellular proliferation in an additive manner (Fig. 6B), suggesting serotonin and IL-1β may mediate VSMC proliferation through both common and independent pathways. To clarify the relationship among IL-1β, HIOMT, and serotonin, we took advantage of *HIOMT* transgenic VSMCs. Compared with wild-type VSMCs, transgenic cells had reduced proliferation in response to IL-1β (Fig. 6C). However, in the presence of both IL-1β and serotonin, wild-type and transgenic cells proliferated to a similar degree (Fig. 6C), indicating serotonin can overcome HIOMT’s proliferation-reducing effect in the presence of IL-1β. These results support the concept that serotonin-mediated VSMC proliferation is independent of 5-MTP or HIOMT.

### Serotonin reduces expression of VSMC contractile markers via the ERK1/2 pathway that is unresponsive to 5-MTP

Synthetic VSMC phenotype, characterized by downregulated VSMC contractile markers, is a prerequisite for proliferation. Indeed, serotonin significantly decreased VSMC markers, including SM-MHC, SM22α, and SM α-actin, in a dose-dependent manner (Additional file 2: Fig. S10). Interestingly, 5-MTP did not rescue serotonin-elicited downregulation of VSMC markers (Fig. 6D). MAPK pathway analysis revealed that serotonin activated ERK1/2, but not p38, in wild-type and transgenic VSMCs to a similar degree (Fig. 6E), suggesting overexpression of *HIOMT373* did not mitigate serotonin-induced ERK1/2 activation. In contrast, IL-1β activated ERK1/2 and p38 in wild-type cells, and the activation was reduced in transgenic cells (Fig. 6E). Intriguingly, inhibiting ERK1/2 activation by U0126 abrogated the reduction of VSMC markers by serotonin (Fig. 6F), indicating the downregulation of VSMC markers by serotonin is mediated via ERK1/2 signaling.

### 5-MTP or *hHIOMT373* overexpression decreases aromatic L-amino acid decarboxylase levels in VSMCs

**Fig. 7:**
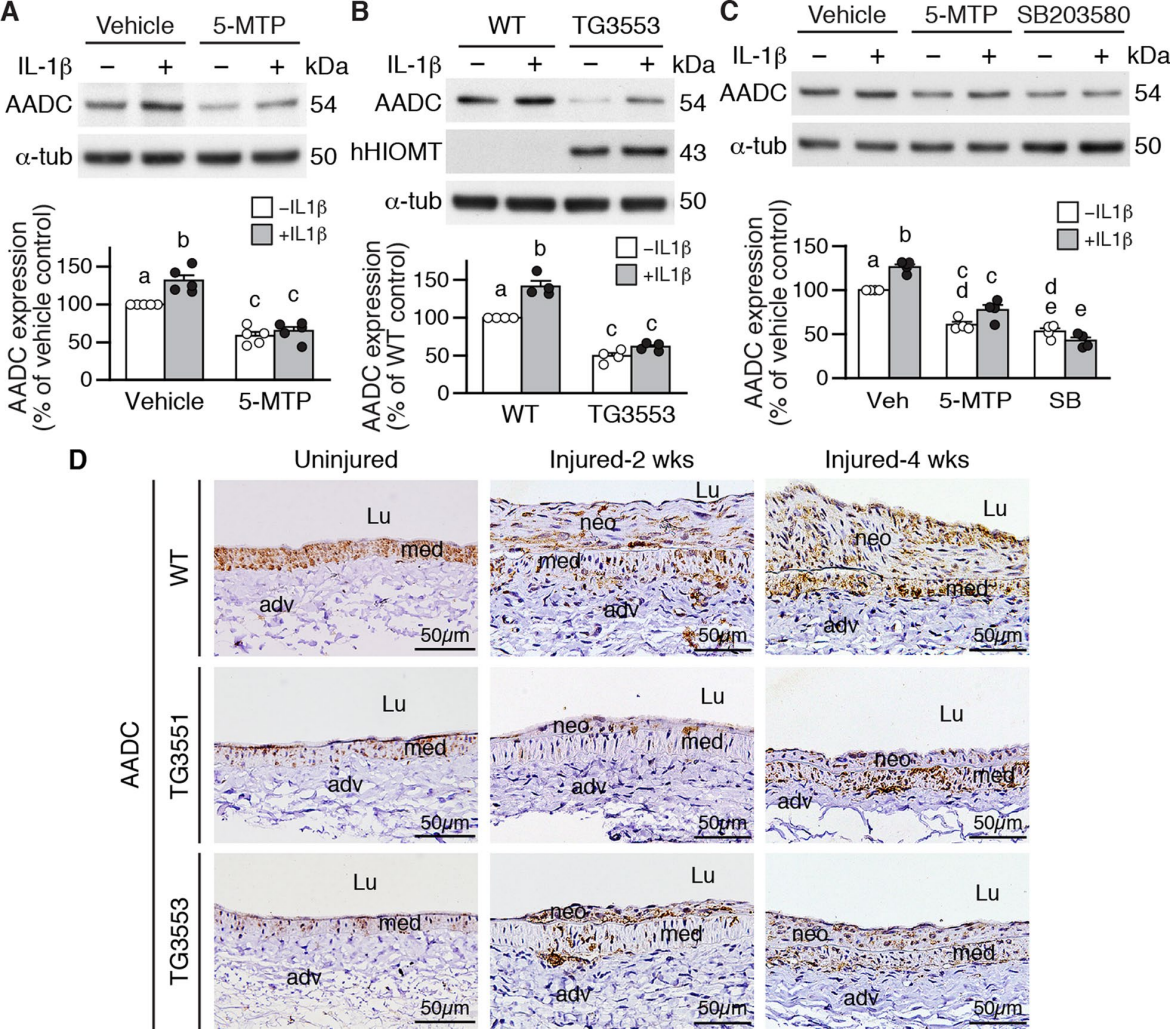
Overexpression of *hHIOMT* decreases AADC levels both in vitro and in vivo. **A** Wild-type (WT) VSMCs were stimulated with vehicle or 5-MTP (100 µmol/L) in the absence or presence of IL-1β (10 ng/mL). Proteins were prepared 24 h later for Western blotting to detect AADC and α-tubulin (α-tub) as a loading control. A representative of 4 experiments is shown. **B** WT and TG3553 VSMCs were treated as in (**A**) and Western blotting was performed to detect AADC, hHIOMT, and α-tubulin (α-tub). A representative is shown, n = 4 each. **C** WT VSMCs were stimulated with vehicle, 5-MTP, or SB203580 in the absence or presence of IL-1β. Proteins were prepared 24 h later for Western blotting to detect AADC and α-tubulin (α-tub) as a loading control. A representative of 4 experiments is shown. **D** AADC immunostaining (brown) of uninjured (WT n = 6; TG3551 n = 2; TG3553 n = 4), 2-week (WT n = 3; TG3551 n = 2; TG3553 n = 2), and 4-week injured (WT n = 5; TG3551 n = 4; TG3553 n = 3) femoral arteries of WT and transgenic mice. Representative sections are shown. Scale bar, 50 µm. Lu, lumen; adv, adventitia; med, media. Different letters indicate significant differences between groups as determined by one-way ANOVA followed by Tukey's test

AADC catalyzes the final step of serotonin synthesis. We confirmed by Western blotting that AADC proteins were detected in untreated VSMCs and the protein level was increased by IL-1β (Fig. 7A). IL-1β-induced AADC upregulation contributed to VSMC proliferation via serotonin, as AADC knockdown by siRNA abrogated the enhancing effect of IL-1β (Additional file 2: Fig. S11A). In addition, increased level of AADC decreased VSMC contractile protein expression, as forced AADC overexpression by transfection reduced SM-MHC, SM22α, and SM α-actin levels (Additional file 2: Fig. S11B). Compared with vector-transfected cells, IL-1β did not exert an additional effect on the contractile proteins in AADC-overexpressed VSMCs (Additional file 2: Fig. S11B), suggesting that IL-1β suppressed contractile protein expression by AADC upregulation. Given the critical role of AADC in VSMC phenotypic changes, we evaluated the effect of 5-MTP on baseline and IL-1β-induced AADC expression. 5-MTP suppressed baseline and IL-1β-induced AADC expression (Fig. 7A). We reasoned that if HIOMT is the synthetic enzyme of 5-MTP, then *hHIOMT* transgenic VSMCs should exert the same effect as 5-MTP in regulating AADC levels. Indeed, transgenic VSMCs (lines 3551 and 3553) had decreased baseline and IL-1β-induced AADC levels (Additional file 2: Fig. S12 and Fig. 7B). Our previous study showed that 5-MTP mediated its effects on vascular cells predominantly via suppressing p38 MAPK signaling [22]; we thus investigated whether 5-MTP regulated AADC expression via p38. Supporting our hypothesis, 5-MTP suppressed baseline AADC and IL-1β-induced AADC levels, and the p38 inhibitor SB203580 exerted similar effects as 5-MTP (Fig. 7C).

Given that serotonin levels were reduced in transgenic arteries (Fig. 5C) and that AADC is the synthetic enzyme for serotonin, we then evaluated AADC levels in wild-type and transgenic arteries. In uninjured arteries, there were fewer AADC-expressing cells in transgenic than in wild-type mice (Fig. 7D, left column). Compared to transgenic animals, which had smaller neointima and fewer AADC-positive cells in the media and neointima, wild-type mice showed an increase in AADC-positive cells in the media and neointima two weeks after vascular injury (Fig. 7D, middle column). Four weeks after injury, neointima grew larger in wild-type mice with many AADC-positive cells. Compared with wild-type mice, transgenic mice had limited growth of neointima and maintained fewer AADC-positive cells than wild-type mice in the media and neointima (Fig. 7D, right column). Collectively, our data suggest that via suppressing p38 activation, 5-MTP inhibits AADC upregulation, thereby decreasing serotonin levels. As such, serotonin appears to function downstream of HIOMT373 and 5-MTP.

## Discussion

A key finding of this study is that HIOMT, which has a central role in regulating circadian rhythms, plays critical functions in VSMCs to protect against vascular injury and intimal hyperplasia. The human functional isoform in VSMCs is the full-length isoform of 373 amino acids. The protective effect of hHIOMT373 overexpression is comparable to that of 5-MTP administration. We have previously reported that 5-MTP possesses potent actions on protecting blood vessels from mechanical and occluded vascular injury [22, 23]. Overexpression of *hHIOMT373* in VSMCs exerts a dual effect on vasoprotection: enhancing the synthesis of vasoprotective 5-MTP and suppressing AADC to lessen the serotonin content. Our results suggest the therapeutic potential of HIOMT/5-MTP for diverse vascular disorders.

The quest for unveiling the identity of COX-2-suppressing cytoguardin in human fibroblasts [35] led to the discovery that 5-MTP, a tryptophan metabolite, was the protective molecule pursued [3, 36]. In the meantime, HIOMT was found to be responsible for the synthesis of 5-MTP from 5-hydroxytryptophan [3]. In mammals, HIOMT was initially identified in the pineal gland and retina for melatonin biosynthesis from N-acetyl-5-hydroxytryptamine (N-acetylserotonin) [1, 37]. HIOMT was later found to be present in a number of peripheral tissues, including the spleen, liver, kidney, heart, and gastrointestinal tract [38, 39]. Interestingly, HIOMT’s functionality decreases in spleen, liver, and heart during physiological aging [38]. These prior studies implicate that in addition to regulating circadian rhythm, HIOMT may have other physiological functions in peripheral tissues. Indeed, HIOMT was found to have a cancer suppressive effect [3, 6]. It is interesting that of the three human HIOMT isoforms, hHIOMT345 is the functional isoform for melatonin generation in pineal cells [2, 5], whereas the active isoform in fibroblasts is hHIOMT298 for 5-MTP production [6]. It was unclear whether VSMCs express HIOMT, nor was it known whether the HIOMT isoform catalyzes 5-MTP synthesis in VSMCs. Here, we reported for the first time that hHIOMT373 is the active isoform responsible for 5-MTP synthesis in VSMCs. Our results showed that hHIOMT298 was functionally inactive. Thus, different isoforms are used by different cells to synthesize 5-MTP and melatonin. How different cells select the HIOMT isoform for 5-MTP vs. melatonin synthesis is unknown. The structure–function relationships of HIOMT373, 298, and 345 in 5-MTP vs. melatonin synthesis are unclear. Further studies are needed to unravel the isoform expression selectivity and to elucidate the structural basis of the isoform catalytic activities.

It is intriguing that overexpression of *HIOMT373* reduced AADC levels both in vitro and in vivo (Fig. 7). Our results suggest that HIOMT-derived 5-MTP regulates AADC expression. Indeed, *HIOMT373* transgenic VSMCs displayed lower baseline and reduced IL-1β-increased AADC levels. Importantly, p38 MAPK inhibitor SB203580 abrogated IL-1β-elicited increases of AADC, indicating p38 activation was required for AADC induction. As 5-MTP mediates its effects mainly through the p38 MAPK pathway [40] and 5-MTP had a similar effect in suppressing AADC levels, HIOMT373-derived 5-MTP is likely to block AADC induction through mitigating IL-1β-induced p38 activation in VSMCs. This finding supports a previous report that *HIOMT* (*HIOMT298*) overexpression in A549 cells exerts a strong suppressive effect on AADC expression, probably through 5-MTP [6].

Serotonin is synthesized from 5-hydroxytryptophan by AADC and can then be converted to N-acetylserotonin (for subsequent melatonin synthesis) by arylalkylamine N-acetyltransferase (AANAT), which is predominantly expressed in the pineal gland and retina [41]. To date, no AANAT expression in VSMCs has been reported. Thus, the presence of HIOMT and AADC in VSMCs would indicate that they share and compete for the same substrate 5-hydroxytryptophan, to produce 5-MTP and serotonin, respectively. Furthermore, because there is no AANAT to metabolize serotonin further, it is not surprising that 5-MTP and serotonin levels were inversely correlated in VSMCs, suggesting potential interplays of tryptophan metabolites. For occlusive vascular disease, a major contributing factor is the phenotypic modulation of VSMCs from a differentiated, contractile phenotype to a dedifferentiated, synthetic phenotype [42]. We have established previously that 5-MTP suppresses activation of p38 MAPK and NFκB, sustains expression of VSMC markers, and maintains the differentiated phenotype of VSMCs, resulting in attenuated VSMC proliferation and migration into the intimal space [23]. Serotonin has been reported to stimulate bovine aortic SMCs' proliferation, which was synergistically enhanced by low-dose PDGF [43]. In line with the proliferation-promoting effect, we found that serotonin decreased VSMC markers via the ERK1/2 pathway (Fig. 6). These results indicate that 5-MTP and serotonin mediate opposing effects on vascular functions.

**Fig. 8:**
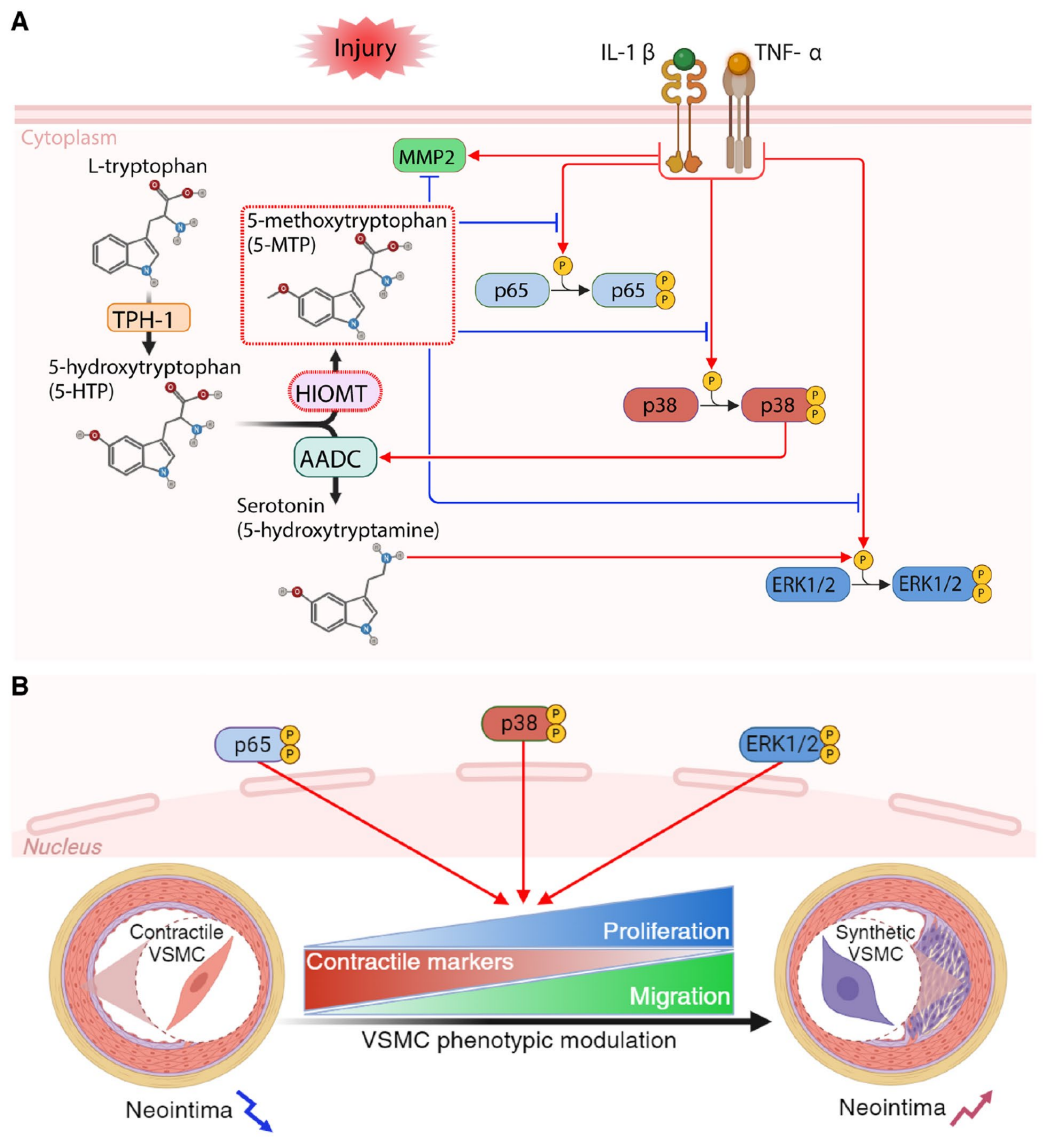
Graphic illustration of how HIOMT-catalyzed 5-MTP via p38 MAPK regulates cytokine-induced AADC expression and serotonin production in VSMCs. **A** TPH-1 catalyzes the conversion of L-tryptophan to 5-HTP, which is a common substrate for serotonin and 5-MTP synthesis catalyzed by AADC and HIOMT, respectively. Artery injury releases cytokines, notably IL-1β and TNF-α, which upregulate AADC expression via p38 MAPK activation, resulting in serotonin overproduction. Serotonin promotes VSMC proliferation and phenotypic switch via the ERK1/2 signaling pathway. Overexpression of HIOMT or 5-MTP administration protects against cellular injury by suppressing AADC expression and thereby serotonin production via inactivation of the p38 MAPK signaling pathway. Thus, HIOMT overexpression in cells shunts the TPH-derived 5-HTP towards 5-MTP synthesis. **B** Collectively, activated p38, ERK1/2, and p65 signaling lead to phenotypic modulation of VSMCs to a synthetic phenotype, resulting in intimal hyperplasia. Inhibition of p38, ERK1/2, and p65 signaling by HIOMT/5-MTP maintains VSMCs in a differentiated phenotype and mitigates neointimal formation. 5-HT P, 5-hydroxytryptophan; 5-MTP, 5-methoxytryptophan; AADC, aromatic L-amino acid decarboxylase; HIOMT, hydroxyindole O-methyltransferase; MMP2, matrix metalloproteinase 2; TPH-1, tryptophan hydroxylase-1; VSMC(s), vascular smooth muscle cell(s). Created with BioRender.com

In summary, injury and inflammatory cytokines (IL-1β and TNF-α) activate p38 MAPK, ERK1/2, and p65 pathways and increase MMP2 expression in VSMCs (Fig. 8A). Activation of p38 increases AADC expression, converting 5-hydroxytryptophan to serotonin, which decreases VSMC markers and increases VSMC proliferation through the ERK1/2 pathway. Our earlier study showed that IL-1β or TNF-α induces VSMC proliferation via the p38 and ERK1/2 signaling pathway, while 5-MTP mitigates the proliferative effect by blocking this pathway [22]. Collectively, these processes lead to phenotypic modulation of VSMCs to a proliferative and migratory synthetic phenotype, resulting in intimal hyperplasia (Fig. 8B). On the other hand, we identified that *hHIOMT373* is the active isoform in VSMCs. Overexpression of *hHIOMT373* diverts 5-hydroxytryptophan to the synthesis of 5-MTP, which decreases MMP2 expression and phosphorylation of p38, ERK1/2, and p65. By inhibiting p38 activation, 5-MTP abrogates AADC induction and decreases serotonin levels. Together, these events maintain VSMCs in a differentiated phenotype and mitigate neointimal formation. Our results demonstrate that HIOMT373 mediates vascular protection via reprogramming tryptophan metabolism by increasing 5-MTP and decreasing serotonin in VSMCs.

## Conclusions

HIOMT, initially identified in the pineal gland for melatonin biosynthesis, was later found in peripheral tissues. We demonstrate for the first time that, in contrast to pineal cells and fibroblasts, hHIOMT373 is the active isoform in VSMCs and protects against intimal hyperplasia. Overexpression of *hHIOMT373* increases 5-MTP production, which mitigates p38 MAPK activation and abrogates the induction of AADC and serotonin, thereby maintaining VSMC differentiated phenotype and preventing vessel occlusion. The previously unrecognized vascular protective function of HIOMT is mediated via reprogramming tryptophan metabolism by increasing 5-MTP and decreasing serotonin in VSMCs, suggesting the therapeutic potential of HIOMT/5-MTP for vascular disorders.

## Supplementary Information

**Supplementary Material 1:** List of antibodies used.

**Supplementary Material 2: Fig. S1.** Schema for the generation of SMC-specific hHIOMT373 transgenic mice. For the conditional transgenic construct, a loxP-emGFP-polyA-NeoR-loxP DNA fragment and a Flag-tagged-hHIOMT373-polyA DNA fragment were inserted into the mouse Actb (beta-actin) intron 1 and exon 2 of the Actb gene, respectively, through DNA recombination in the bacterial artificial chromosome. The construct was then used to generate Actb-emGFP-hHIOMT373^WT/flox^ conditional transgenic mice (Actb-emGFP), which were then bred with SM22α-Cre mice to generate SMC-hHIOMT373 transgenic mice. **Fig. S2.** Different tissues of Actb-emGFP-hHIOMT373 mice do not express the hHIOMT373 transgene. **A** Total proteins from the aorta and femoral artery (FA) from wild-type (WT) and Actb-emGFP-hHIOMT373 mice were subjected to Western blot analysis to detect hHIOMT, emGFP, and GAPDH as a loading control. WT served as a negative control, while the aortic proteins from the global hHIOMT373 transgenic mice served as a positive control for hHIOMT transgene expression. **B** Total proteins were isolated from the heart and kidney from 2 different WT and 2 different Actb-emGFP-hHIOMT373 mice. Western blot analysis was performed to detect hHIOMT, emGFP, and GAPDH as a loading control. WT served as a negative control. The heart proteins from the global hHIOMT373 transgenic mice served as a positive control for hHIOMT transgene expression. **Fig. S3**. Pathological conditions induce hHIOMT and 5-MTP levels in HASMCs and in human arteriosclerotic arteries. **A** HASMCs were treated with or without IL-1β (10 ng/mL) for 72 h, and RNA was isolated for qRT-PCR analysis of *hHIOMT* expression. Gene expression was normalized to human *GAPDH* and analyzed using the 2^−ΔΔCt^ method. Data are shown as mean ± SEM (n = 4 each). *P < 0.05 (unpaired Student’s t-test). **B** HASMCs were treated with or without IL-1β, and conditioned medium was collected to measure 5-MTP levels by quantitative LC–MS–MS. 5-MTP levels were normalized to cellular RNA amounts and expressed as nM/ng RNA. Data are presented as mean ± SEM (n = 3 each). *P < 0.05 (paired Student’s t-test). **C** Immunohistochemical analysis of hHIOMT and 5-MTP was performed on normal and arteriosclerotic human arterial sections. Brown color indicates positive staining. Tunica media are shown. The inset in each panel shows a magnified image of the tunica media. Scale bar, 200 µm. **Fig. S4.** Expression of hHIOMT373 transgene in different tissues of transgenic mice. Total proteins were isolated from aorta and other tissues from wild-type (WT) and 7 transgenic lines for Western blot analysis. WT served as a negative control. The human Y79 cell line expresses the hHIOMT345 isoform, encoding a 38 kDa protein, and thus Y79 cell extracts served as a positive control for hHIOMT. Blots were hybridized to hHIOMT antibody and GAPDH for loading control. **Fig. S5.** hHIOMT373 transgene and 5-MTP expression in the wild-type and transgenic mouse aortas. **A-B** Immunohistochemistry was performed on aortic sections to detect **A** the hHIOMT373 transgene and **B** 5-MTP expression using hHIOMT and 5-MTP antibodies, respectively (scale bar, 200 µm). The corresponding bottom panel displays a higher magnification of the boxed area (scale bar, 50 µm). Lu, lumen; med, media. **Fig. S6.** Plasma 5-MTP and endogenous and transgene expression in wild-type (WT) and hHIOMT373 transgenic (TG) mice and vascular smooth muscle cells. **A** Blood was drawn from WT (n = 4), the TG mouse line 3551 (TG3551, n = 4), and line 3553 (TG3553, n = 4), and plasma 5-MTP levels were measured by quantitative LC–MS–MS. *P < 0.05 (one-way ANOVA, followed by Tukey’s test). **B** Total RNA was isolated from WT and TG VSMCs, and quantitative RT-PCR was performed to measure endogenous *mHiomt* and exogenous *hHIOMT*. Gene expression level was normalized to the mouse *Gapdh* using the ∆Ct method. Data was presented as mean ± SEM (n = 4). ****P < 0.0001 (unpaired Student’s *t*-test). **Fig. S7.** Vascular smooth muscle-specific expression of hHIOMT (SMC-*hHIOMT*) in mice increases 5-MTP levels in the medial layer and endothelium but does not change HIOMT levels in the endothelium. Femoral arteries from wild-type (WT), Actb-emGFP, and SMC-hHIOMT mice were harvested and sectioned. **A** HIOMT expression was detected by immunostaining arterial sections with hHIOMT antibody (brown). HIOMT was only detected in the medial layer but not in the endothelium of the SMC-*hHIOMT* arteries. **B** Immunostaining by 5-MTP antibody detected a baseline expression of 5-MTP (brown) in the medial smooth muscle cells and endothelial cells from WT and Actb-emGFP arteries. High levels of 5-MTP expression were detected in the medial layer and endothelium from SMC-*hHIOMT* mice. Arrows indicate endothelial cells (EC). med, media; adv, adventitia. Scale bar, 25 µm. **Fig. S8.** Vascular smooth muscle-specific expression of hHIOMT in mice attenuates intimal hyperplasia following arterial injury. **A** Actb-emGFP line 110 (n = 6) and SMC-hHIOMT line 110 mice (n = 6) were subjected to femoral artery denudation injury. Vessels were harvested 4 weeks after injury for histological analysis. Verhoeff’s elastin stain was performed on femoral artery longitudinal sections (scale bar, 100 µm). Higher magnification of the boxed area is shown in the respective bottom panel (scale bar, 50 µm). Representative images are shown. Lu, lumen; neo, neointima; adv, adventitia; med, media. **B** The intima-to-media ratio was calculated. **P < 0.01 (two-tailed, unpaired Student’s *t*-test). **Fig. S9.** HIOMT expression reduces arterial MMP2 levels after injury and inflammatory cytokine-induced VSMC inflammation, proliferation, and migration. **A** MMP2 immunostaining (brown) was performed on control and 2-week injured femoral arterial sections from wild-type (WT, n = 3) and transgenic (TG, n = 3) mice. Representative sections are shown. adv, adventitia; med, media; neo, neointima; EEL, external elastic lamina. Scale bar, 25 µm. **B** WT and TG VSMCs were stimulated with or without IL-1β for 24 h, and proteins were harvested for Western blotting to detect MMP2, hHIOMT373, and α-tubulin for loading control. A representative of 3 experiments is shown. **C** VSMCs were plated, serum-starved, and then stimulated with or without IL-1β for 15 min. Proteins were prepared for Western blotting to detect phosphorylated and total p38 and p65, hHIOMT373, or α-tubulin for loading control. A representative of 4 experiments is shown. **D** Cells were stimulated with vehicle, PDGF-BB, IL-1β, or TNF-α for 24 h, and proliferation was assessed by CCK-8 kit. n = 4 each. Different letters indicate significant differences between groups as determined by one-way ANOVA followed by Tukey's test. **E** VSMCs were plated, wounded with a p200 tip, and then treated with vehicle, 10 ng/mL of PDGF-BB, IL-1β, or TNF-α in starvation medium for 6 h. Wound images were captured at time 0 and at 6 h. Wound closure was quantified, and % wound closure was calculated. n = 4 each. Different letters indicate significant differences between groups as determined by one-way ANOVA followed by Tukey's test. **Fig. S10.** Serotonin dose-dependently reduces VSMC marker expression. WT VSMCs were treated with increasing concentrations of serotonin. Proteins were prepared 24 h later for Western blotting to detect SM-MHC, SM22α, SM α-actin, and α-tubulin as a loading control. A representative of 3 experiments is shown. Quantitative analysis of expression levels of VSMC markers. *P < 0.05 vs. control without serotonin of the respective VSMC marker (one-way ANOVA followed by Dunnett’s test). **Fig. S11.** AADC knockdown suppresses IL-1β-induced VSMC proliferation, while AADC overexpression reduces VSMC contractile marker expression. **A** VSMCs were transfected with 20 nmol/L control siRNA (siControl) or AADC siRNA (siAADC) and then stimulated with or without serotonin and/or IL-1β. Proliferation was then assessed by CCK-8 assays 24 h later. n = 4 each. **B** Wild-type VSMCs were transfected with pCMV6-GFP vector or pCMV6-AADC expression plasmid and then treated with or without IL-1β. Proteins were prepared 24 h later for Western blotting to detect SM-MHC, SM22α, SM α-actin, and α-tubulin as a loading control. A representative of 4 experiments is shown (left panel). Quantitative analysis of expression levels of VSMC markers (right panels). Different letters indicate significant differences between groups as determined by one-way ANOVA followed by Tukey's test. **Fig. S12.** Overexpression of hHIOMT decreases AADC levels in VSMCs. Primary cultured wild-type and hHIOMT transgenic (TG3551 line) VSMCs were serum-starved and then treated with or without IL-1β (10 ng/mL). Proteins were prepared 24 h later for Western blotting to detect AADC, hHIOMT, and α-tubulin as a loading control. A representative of 4 experiments is shown. Different letters indicate significant differences between groups as determined by one-way ANOVA followed by Tukey’s test.

## Data Availability

All data generated or analyzed during this study are included in this published article and its supplementary information files.

## Abbreviations

5-MTP: 5-Methoxytryptophan
AADC: Aromatic L-amino acid decarboxylase
AANAT: Arylalkylamine N-acetyltransferase
ASMT: N-acetylserotonin methyltransferase
*Actb*: Mouse beta-actin gene
BAC: Bacterial artificial chromosome
BSA: Bovine serum albumin
CM: Conditioned medium
HIOMT: Hydroxyindole O-methyltransferase
hHIOMT: Human hydroxyindole O-methyltransferase
IL-1β: Interleukin-1 beta
MMP2: Matrix metalloproteinase 2
NFκB: Nuclear factor kappa B
PDGF-BB: Platelet-derived growth factor-BB
SM 22α: Smooth muscle 22 alpha
SM α-actin: Smooth muscle alpha-actin
SMC(s): Smooth muscle cell(s)
SM-MHC: Smooth muscle-myosin heavy chain
TPH-1: Tryptophan hydroxylase-1
TNF-α: Tumor necrosis factor-alpha
VSMC(s): Vascular smooth muscle cell(s)
