# Supplementary material for: Smooth muscle-specific expression of hydroxyindole O-methyltransferase reduces arterial injury-induced intimal hyperplasia
Source: J Biomed Sci. 2025 Aug 20;32:78. doi: 10.1186/s12929-025-01172-4 (PMC12366072; doi:10.1186/s12929-025-01172-4)
Supplement: Supplementary file 1 — Supplementary Material 1: List of antibodies used. [file 12929_2025_1172_MOESM1_ESM.pdf]

## Western blot analysis

| Primary antibody | Vendor | Cat. Number | Secondary antibody | Vendor | Cat. Number |
|---|---|---|---|---|---|
| p38MAPK | Cell Signaling | 9212 | Goat anti-Rabbit | Thermo | 31460 |
| p-p38MAPK | Cell Signaling | 9211 | Goat anti-Rabbit | Thermo | 31460 |
| AADC (DOPA decarboxylase) | Abcam | Ab3905 | Goat anti-Rabbit | Thermo | 31460 |
| ASMT/HIOMT (human) | Abcam | ab180511 | Goat anti-Rabbit | Thermo | 31460 |
| ERK1/2 | Cell Signaling | 9102 | Goat anti-Rabbit | Thermo | 31460 |
| p-ERK1/2 | Cell Signaling | 9106 | Goat anti-Mouse | Millipore | AP124P |
| Flag | Cell Signaling | 14793 | Goat anti-Rabbit | Thermo | 31460 |
| GAPDH | GeneTex | GTX627408 | Goat anti-Mouse | Millipore | AP124P |
| GFP | Takara | 632381 | Goat anti-Mouse | Millipore | AP124P |
| MMP2 | Abcam | ab37150 | Goat anti-Rabbit | Thermo | 31460 |
| NFκB/p65 | Cell Signaling | 6956 | Goat anti-Mouse | Millipore | AP124P |
| p-NFkB/p65 | Cell Signaling | 3033 | Goat anti-Rabbit | Thermo | 31460 |
| SM22α (Transgelin) | Abcam | ab14106 | Goat anti-Rabbit | Thermo | 31460 |
| SM α-actin | Sigma-Aldrich | A5228 | Goat anti-Mouse | Millipore | AP124P |
| SM-MHC | Proteintech | 21404-1-AP | Goat anti-Rabbit | Thermo | 31460 |
| α-Tubulin | Cell Signaling | 3873 | Goat anti-Mouse | Millipore | AP124P |

## Immunohistochemistry

| Primary antibody | Vendor | Cat. Number | Secondary antibody | Vendor | Cat. Number |
|---|---|---|---|---|---|
| AADC (DOPA decarboxylase) | Abcam | ab3905 | Goat anti-Rabbit | DAKO | K4003 |
| ASMT/HIOMT (human) | Abcam | ab180511 | Goat anti-Rabbit | DAKO | K4003 |
| Flag | Cell Signaling | 14793 | Goat anti-Rabbit | DAKO | K4003 |
| GFP | Takara | 632381 | Goat anti-Mouse | DAKO | K4001 |
| MMP2 | Abcam | ab37150 | Goat anti-Rabbit | DAKO | K4003 |
| 5-MTP | GenScript | Custom-made | Goat anti-Rabbit | DAKO | K4003 |
| Serotonin | Abcam | ab66047 | Donkey anti-Goat | Jackson | 705035003 |
| SM α-actin | Sigma-Aldrich | A5528 | Goat anti-Mouse | DAKO | K4001 |

## Immunofluorescence

| Primary antibody | Vendor | Cat. Number | Secondary antibody | Vendor | Cat. Number |
|---|---|---|---|---|---|
| ASMT/HIOMT (human) | Abcam | ab180511 | donkey anti-rabbit IgG (H+L), Alexa Fluor™ 594 | Invitrogen | A21207 |
| 5-MTP | GenScript | Custom-made | donkey anti-rabbit IgG (H+L), Alexa Fluor™ 488 | Invitrogen | A21206 |
| Flag | Sigma-Aldrich | F3165 | donkey anti-mouse IgG (H&L), Alexa Fluor™ 488 | Invitrogen | A-21202 |
| 5-MTP | GenScript | Custom-made | Goat Anti-Rabbit IgG H&L (Alexa Fluor® 594) | Abcam | ab150080 |
| Serotonin | Abcam | ab66047 | Donkey Anti-Goat IgG H&L (Alexa Fluor® 488) | Abcam | ab150129 |
