# Supplementary material for: Smooth muscle-specific expression of hydroxyindole O-methyltransferase reduces arterial injury-induced intimal hyperplasia
Source: J Biomed Sci. 2025 Aug 20;32:78. doi: 10.1186/s12929-025-01172-4 (PMC12366072; doi:10.1186/s12929-025-01172-4)
Supplement: Supplementary file 2 — Supplementary Material 2: Fig. S1. Schema for the generation of SMC-specific hHIOMT373 transgenic mice. For the conditional transgenic construct, a loxP-emGFP-polyA-NeoR-loxP DNA fragment and a Flag-tagged-hHIOMT373-polyA DNA fragment were inserted into the mouse Actb (beta-actin) intron 1 and exon 2 of the Actb gene, respectively, through DNA recombination in the bacterial artificial chromosome. The construct was then used to generate Actb-emGFP-hHIOMT373WT/flox conditional transgenic mice (Actb-emGFP), which were then bred with SM22α-Cre mice to generate SMC-hHIOMT373 transgenic mice. Fig. S2. Different tissues of Actb-emGFP-hHIOMT373 mice do not express the hHIOMT373 transgene. A Total proteins from the aorta and femoral artery (FA) from wild-type (WT) and Actb-emGFP-hHIOMT373 mice were subjected to Western blot analysis to detect hHIOMT, emGFP, and GAPDH as a loading control. WT served as a negative control, while the aortic proteins from the global hHIOMT373 transgenic mice served as a positive control for hHIOMT transgene expression. B Total proteins were isolated from the heart and kidney from 2 different WT and 2 different Actb-emGFP-hHIOMT373 mice. Western blot analysis was performed to detect hHIOMT, emGFP, and GAPDH as a loading control. WT served as a negative control. The heart proteins from the global hHIOMT373 transgenic mice served as a positive control for hHIOMT transgene expression. Fig. S3. Pathological conditions induce hHIOMT and 5-MTP levels in HASMCs and in human arteriosclerotic arteries. A HASMCs were treated with or without IL-1β (10 ng/mL) for 72 h, and RNA was isolated for qRT-PCR analysis of hHIOMT expression. Gene expression was normalized to human GAPDH and analyzed using the 2−ΔΔCt method. Data are shown as mean ± SEM (n = 4 each). *P < 0.05 (unpaired Student’s t-test). B HASMCs were treated with or without IL-1β, and conditioned medium was collected to measure 5-MTP levels by quantitative LC–MS–MS. 5-MTP levels were normalized to ce [file 12929_2025_1172_MOESM2_ESM.pdf]

Figure S1

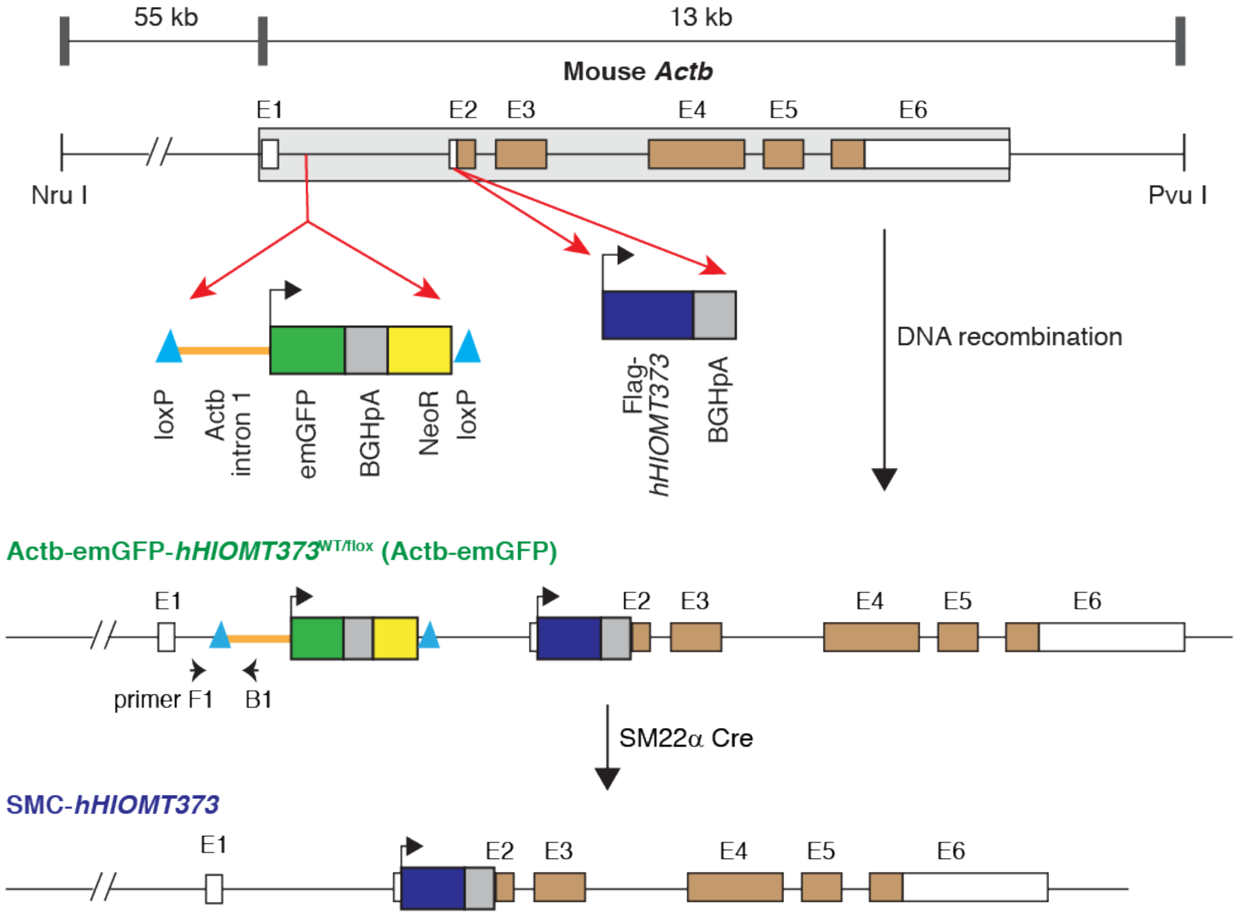

Fig. S1. Schema for the generation of SMC-specific hHIOMT373 transgenic mice. For the conditional transgenic construct, a loxP-emGFP-polyA-NeoR-loxP DNA fragment and a Flag-tagged-*hHIOMT373*-polyA DNA fragment were inserted into the mouse *Actb* (beta-actin) intron 1 and exon 2 of the *Actb* gene, respectively, through DNA recombination in the bacterial artificial chromosome. The construct was then used to generate Actb-emGFP-*hHIOMT373*$^{WT/flox}$ conditional transgenic mice (Actb-emGFP), which were then bred with SM22α-Cre mice to generate SMC-*hHIOMT373* transgenic mice.

Figure S2

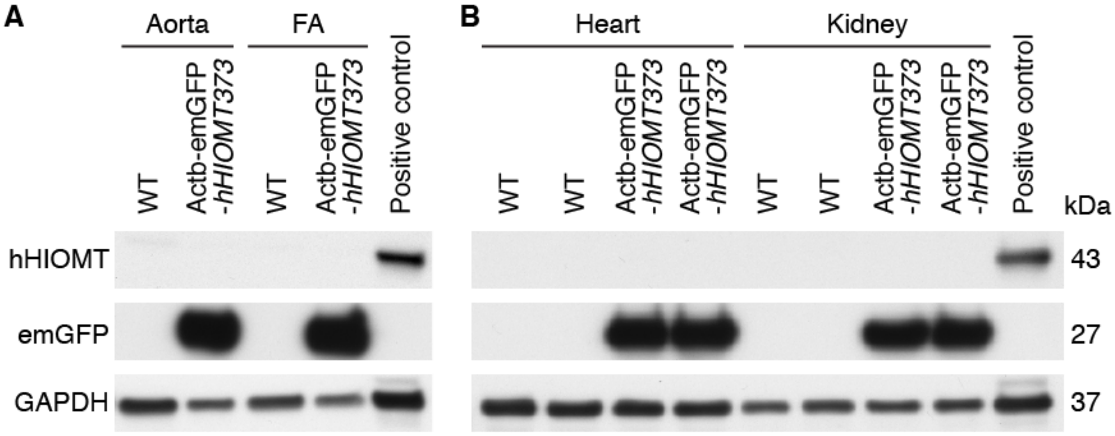

Fig. S2. Different tissues of Actb-emGFP-*hHIOMT373* mice do not express the hHIOMT373 transgene. **A** Total proteins from the aorta and femoral artery (FA) from wild-type (WT) and Actb-emGFP-*hHIOMT373* mice were subjected to Western blot analysis to detect hHIOMT, emGFP, and GAPDH as a loading control. WT served as a negative control, while the aortic proteins from the global *hHIOMT373* transgenic mice served as a positive control for hHIOMT transgene expression. **B** Total proteins were isolated from the heart and kidney from 2 different WT and 2 different Actb-emGFP-*hHIOMT373* mice. Western blot analysis was performed to detect hHIOMT, emGFP, and GAPDH as a loading control. WT served as a negative control. The heart proteins from the global *hHIOMT373* transgenic mice served as a positive control for hHIOMT transgene expression.

Figure S3

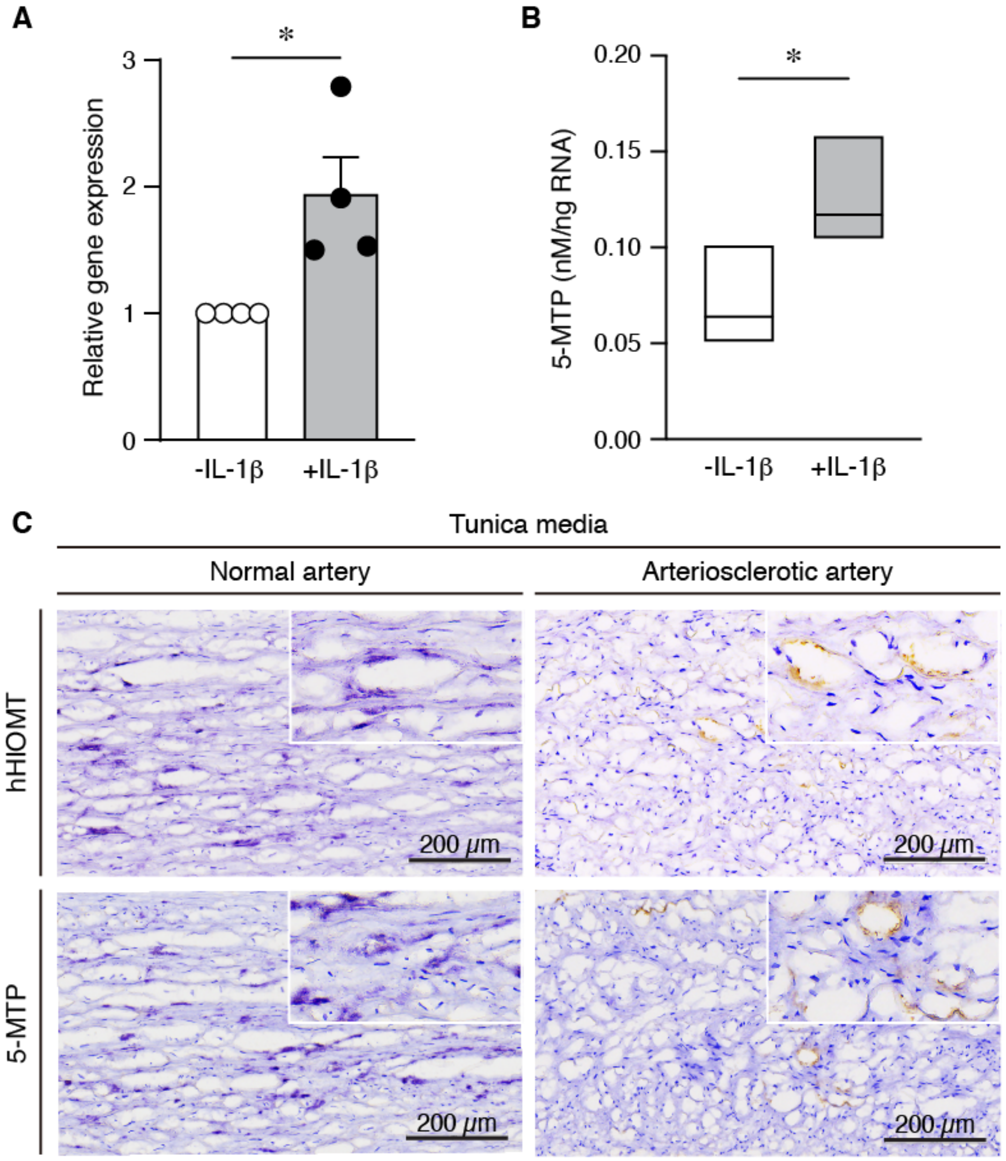

Fig. S3. Pathological conditions induce *hHIOMT* and 5-MTP levels in HASMCs and in the human arteriosclerotic arteries. **A** HASMCs were treated with or without IL-1β (10 ng/mL) for 72 h, and RNA was isolated for qRT-PCR analysis of *hHIOMT* expression. Gene expression was normalized to human *GAPDH* and analyzed using the $2^{-\Delta\Delta Ct}$ method. Data are shown as mean ± SEM (n=4 each). **P*<0.05 (unpaired Student's *t*-test). **B** HASMCs were treated with or without IL-1β, and conditioned medium was collected to measure 5-MTP levels by quantitative LC–MS-MS. 5-MTP levels were normalized to cellular RNA amounts and expressed as nM/ng RNA. Data are presented as mean ± SEM (n=3 each). **P*<0.05 (paired Student's *t*-test). **C** Immunohistochemical analysis of hHIOMT and 5-MTP was performed on normal and arteriosclerotic human arterial sections. Brown color indicates positive staining. Tunica media are shown. The inset in each panel shows a magnified image of the tunica media. Scale bar, 200 μm.

Figure S4

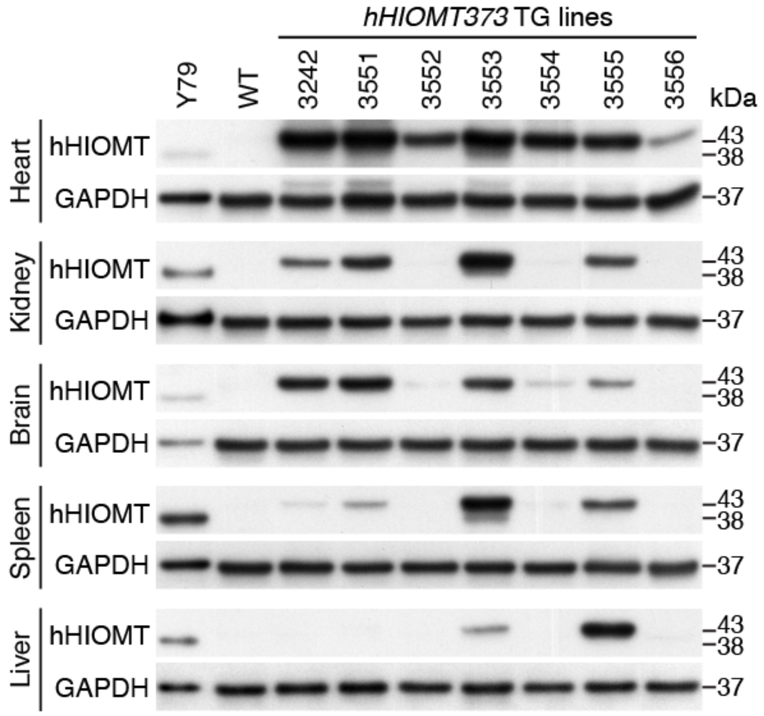

Fig. S4. Expression of hHIOMT373 transgene in different tissues of transgenic mice. Total proteins were isolated from aorta and other tissues from wild-type (WT) and 7 transgenic lines for Western blot analysis. WT served as a negative control. The human Y79 cell line expresses the *hHIOMT345* isoform, encoding a 38 kDa protein, and thus Y79 cell extracts served as a positive control for hHIOMT. Blots were hybridized to hHIOMT antibody and GAPDH for loading control.

Figure S5

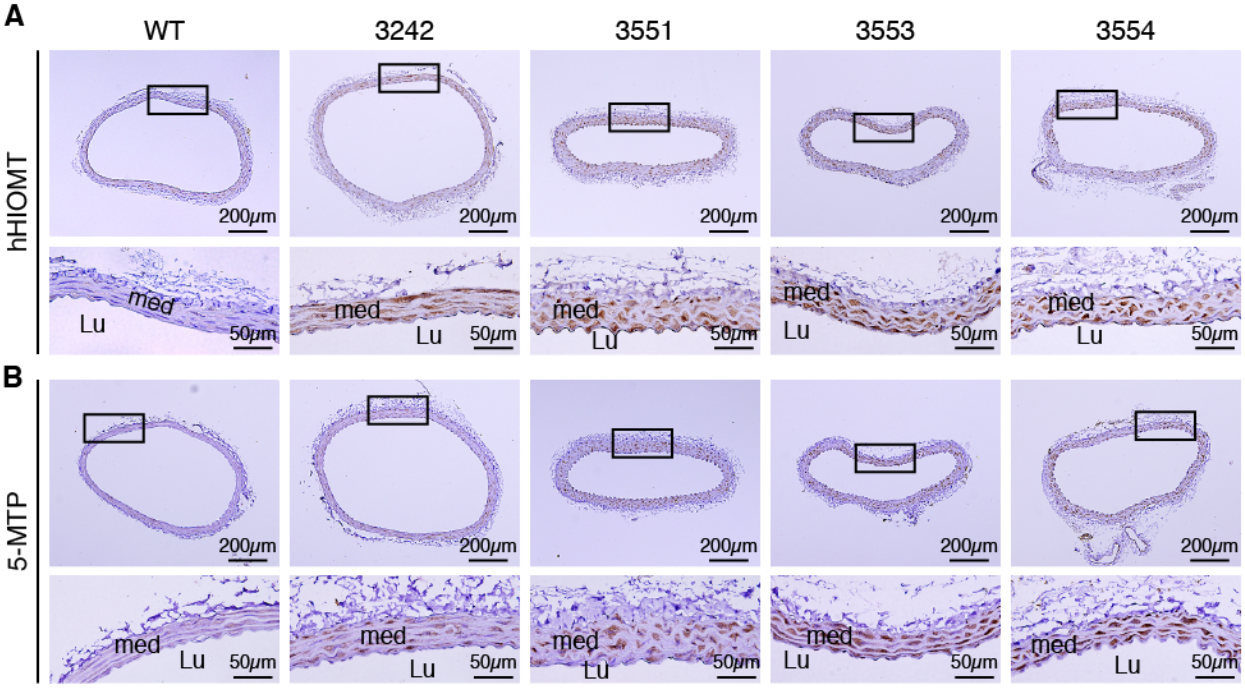

Fig. S5. hHIOMT373 transgene and 5-MTP expression in the wild-type and transgenic mouse aortas. **A-B** Immunohistochemistry was performed on aortic sections to detect **A** the hHIOMT373 transgene and **B** 5-MTP expression using hHIOMT and 5-MTP antibodies, respectively (scale bar, 200 μm). The corresponding bottom panel displays a higher magnification of the boxed area (scale bar, 50 μm). Lu, lumen; med, media.

Figure S6

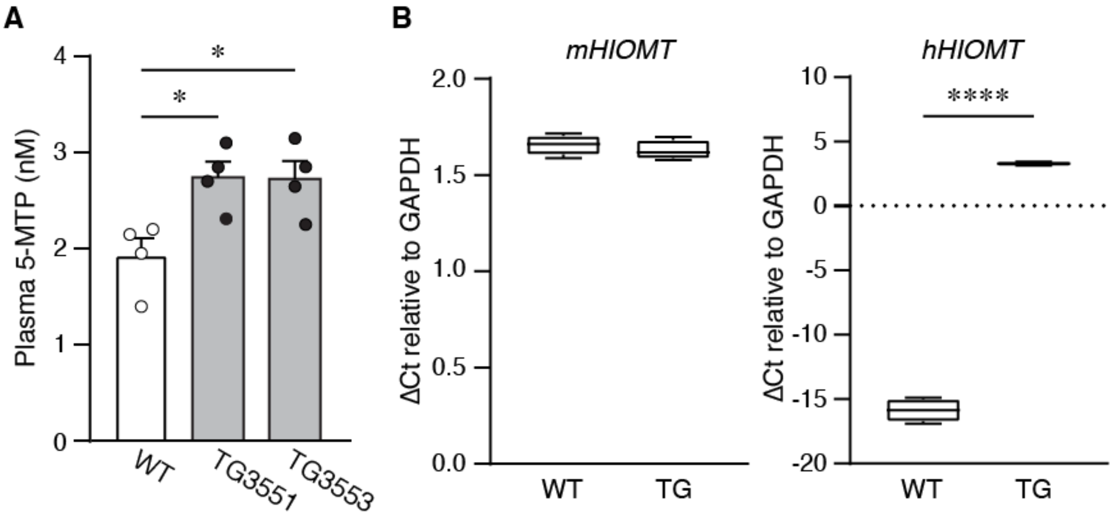

Fig. S6. Plasma 5-MTP and endogenous and transgene expression in wild-type (WT) and hHIOMT373 transgenic (TG) mice and vascular smooth muscle cells. **A** Blood was drawn from WT (n=4), the TG mouse line 3551 (TG3551, n=4), and line 3553 (TG3553, n=4), and plasma 5-MTP levels were measured by quantitative LC–MS-MS. **P*<0.05 (one-way ANOVA, followed by Tukey's test). **B** Total RNA was isolated from WT and TG VSMCs, and quantitative RT-PCR was performed to measure endogenous *mHiomt* and exogenous *hHIOMT*. Gene expression level was normalized to the mouse *Gapdh* using the ΔCt method. Data was presented as mean ± SEM (n=4). *****P*<0.0001 (unpaired Student's *t*-test).

Figure S7

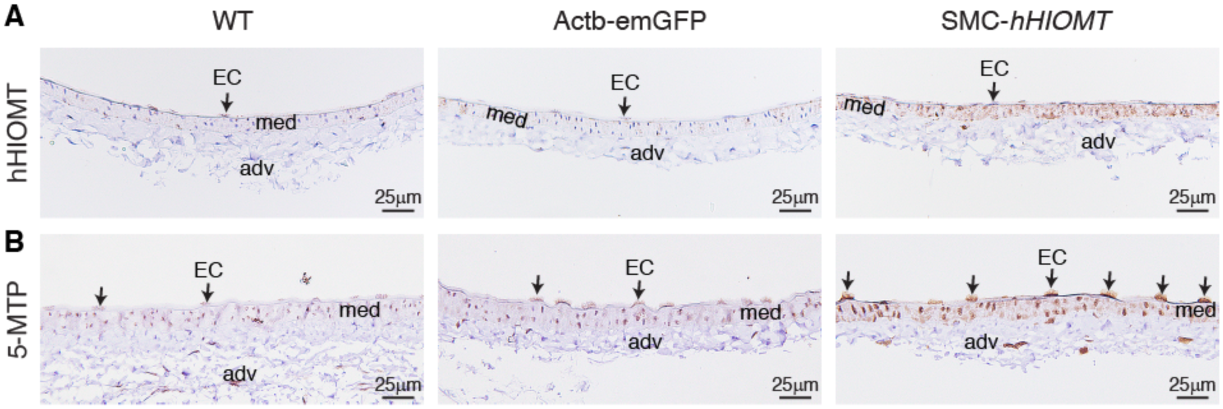

Fig. S7. Vascular smooth muscle-specific expression of hHIOMT (SMC-*hHIOMT*) in mice increases 5-MTP levels in the medial layer and endothelium but does not change HIOMT levels in the endothelium. Femoral arteries from wild-type (WT), Actb-emGFP, and SMC-*hHIOMT* mice were harvested and sectioned. **A** HIOMT expression was detected by immunostaining arterial sections with hHIOMT antibody (brown). HIOMT was only detected in the medium but not in the endothelium of the SMC-*hHIOMT* arteries. **B** Immunostaining by 5-MTP antibody detected a baseline expression of 5-MTP (brown) in the medial smooth muscle cells and endothelial cells from WT and Actb-emGFP arteries. High levels of 5-MTP expression were detected in the medial layer and endothelium from SMC-*hHIOMT* mice. Arrows indicate endothelial cells (EC). med, media; adv, adventitia. Scale bar, 25 μm.

Figure S8

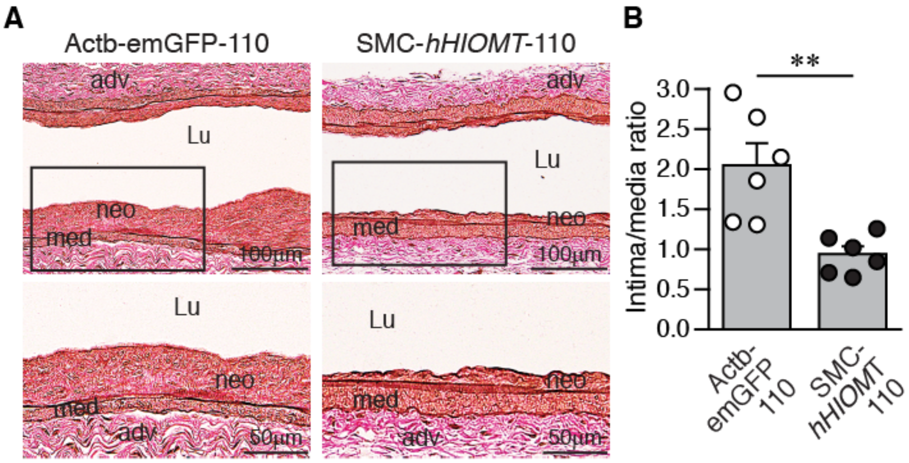

Fig. S8. Vascular smooth muscle-specific expression of hHIOMT in mice attenuates intimal hyperplasia following arterial injury. **A** Actb-emGFP line 110 (n=6) and SMC-*hHIOMT* line 110 mice (n=6) were subjected to femoral artery denudation injury. Vessels were harvested 4 weeks after injury for histological analysis. Verhoeff's elastin stain was performed on femoral artery longitudinal sections (scale bar, 100 μm). Higher magnification of the boxed area is shown at the respective bottom panel (scale bar, 50 μm). Representative images are shown. Lu, lumen; neo, neointima; adv, adventitia; med, media. **B** The intima-to-media ratio was calculated. ***P*<0.01 (two-tailed, unpaired Student's *t*-test).

Figure S9

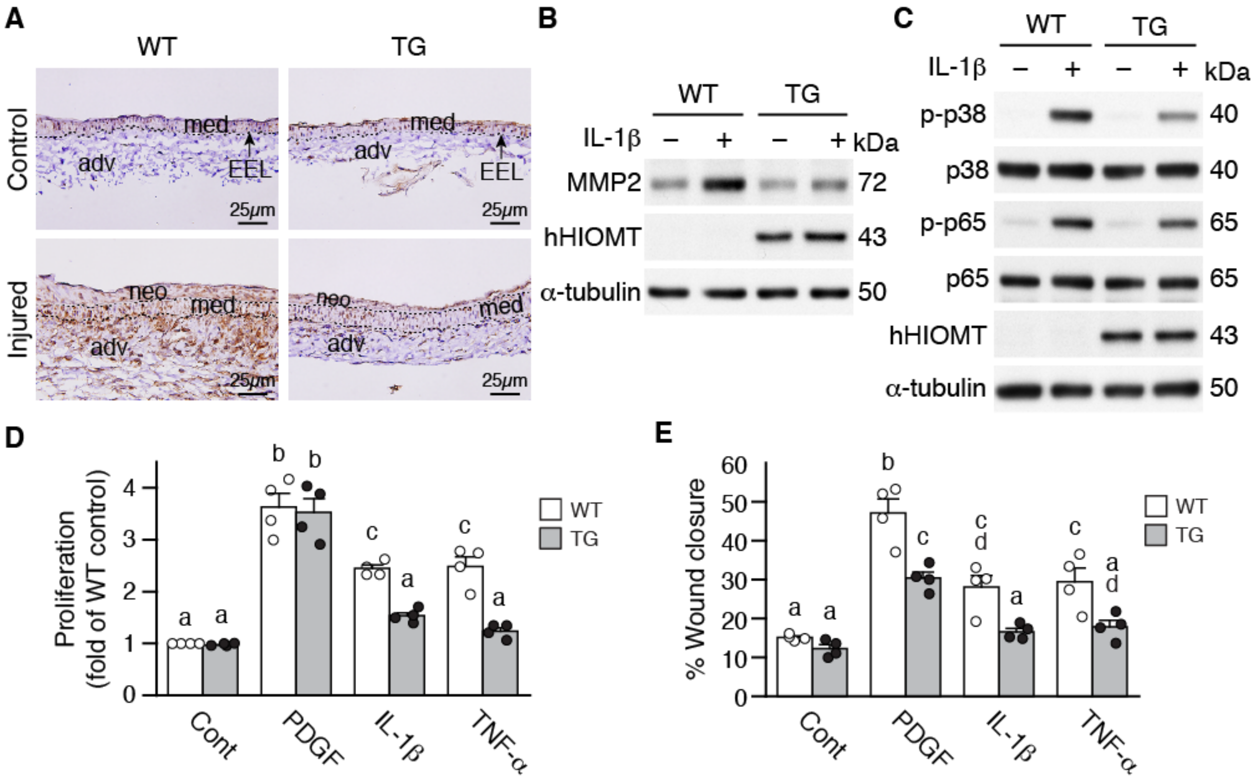

Fig. S9. HIOMT expression reduces arterial MMP2 levels after injury and inflammatory cytokine-induced VSMC inflammation, proliferation, and migration. **A** MMP2 immunostaining (brown) was performed on control and 2-week injured femoral arterial sections from WT (n=3) and TG (n=3) mice. Representative sections are shown. adv, adventitia; med, media; neo, neointima; EEL, external elastic lamina. Scale bar, 25 μm. **B** WT and TG VSMCs were stimulated with or without IL-1β for 24 h, and proteins were harvested for Western blotting to detect MMP2, hHIOMT373, and α-tubulin for loading control. A representative of 3 experiments is shown. **C** VSMCs were plated, serum-starved, and then stimulated with or without IL-1β for 15 min. Proteins were prepared for Western blotting to detect phosphorylated and total p38 and p65, hHIOMT373, or α-tubulin for loading control. A representative of 4 experiments is shown. **D** Cells were stimulated with vehicle, PDGF-BB, IL-1β, or TNF-α for 24 h, and proliferation was assessed by CCK-8 kit. n=4 each. Different letters indicate significant differences between groups as determined by one-way ANOVA followed by Tukey's test. **E** VSMCs were plated, wounded with a p200 tip, and then treated with vehicle, 10 ng/mL of PDGF-BB, IL-1β, or TNF-α in starvation medium for 6 h. Wound images were captured at time 0 and at 6 h. Wound closure was quantified, and % wound closure was calculated. n=4 each. Different letters indicate significant differences between groups as determined by one-way ANOVA followed by Tukey's test.

Figure S10

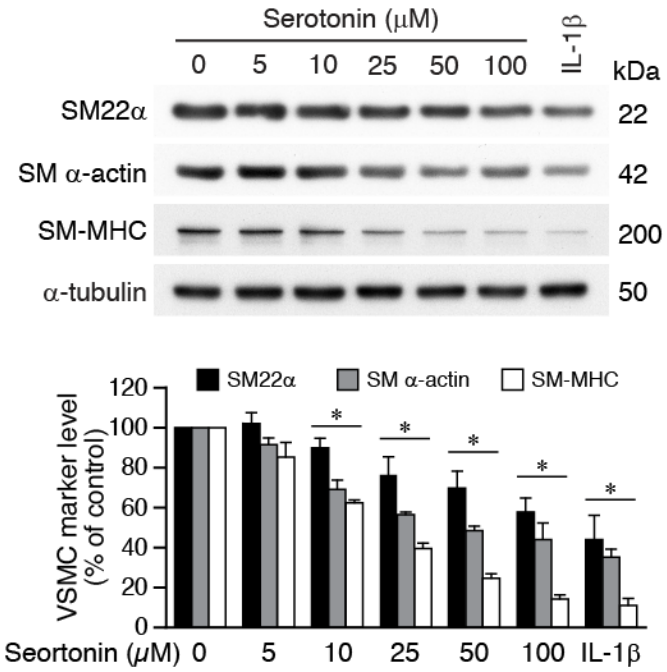

Fig. S10. Serotonin dose-dependently reduces VSMC marker expression. WT VSMCs were treated with increasing concentrations of serotonin. Proteins were prepared 24 h later for Western blotting to detect SM-MHC, SM22α, SM α-actin, and α-tubulin as a loading control. A representative of 3 experiments is shown. Quantitative analysis of expression levels of VSMC markers. **P*<0.05 vs. control without serotonin of the respective VSMC marker (one-way ANOVA followed by Dunnett's test).

Figure S11

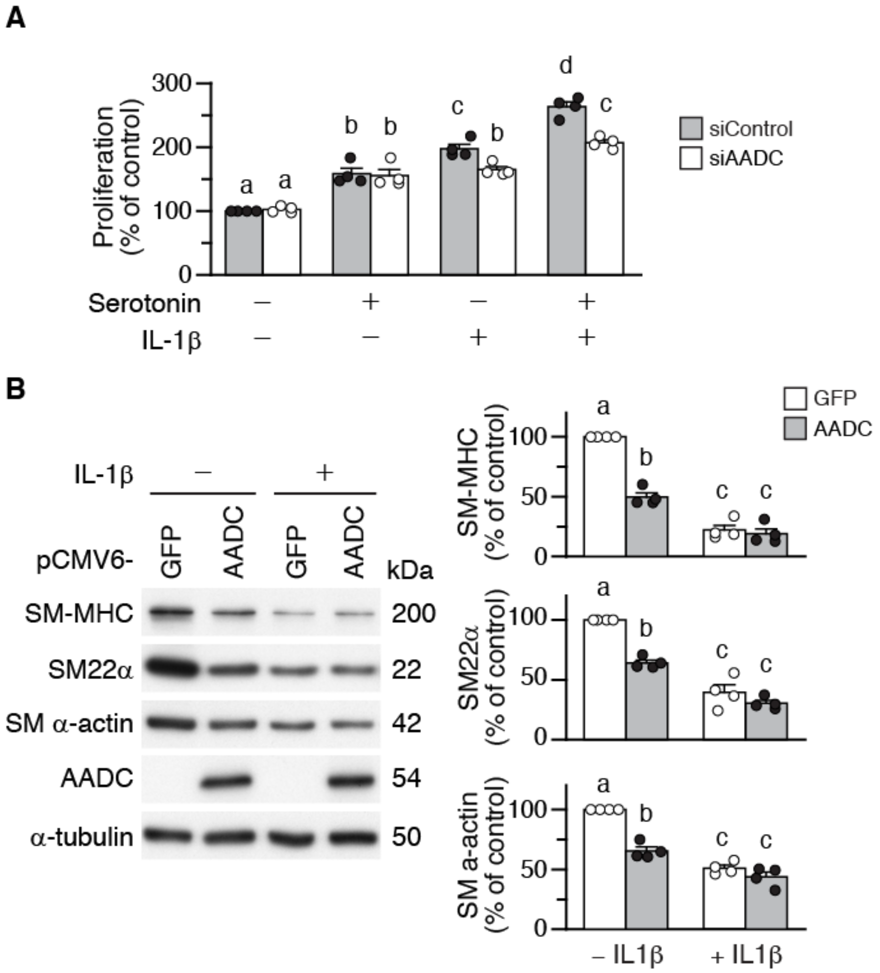

Fig. S11. AADC knockdown suppresses IL-1β-induced VSMC proliferation, while AADC overexpression reduces VSMC contractile marker expression. **A** VSMCs were transfected with 20 nmol/L control siRNA (siControl) or AADC siRNA (siAADC) and then stimulated with or without serotonin and/or IL-1β. Proliferation was then assessed by CCK-8 assays 24 h later. n=4 each. **B** WT VSMCs were transfected with pCMV6-GFP vector or pCMV6-AADC expression plasmid and then treated with or without IL-1β. Proteins were prepared 24 h later for Western blotting to detect SM-MHC, SM22α, SM α-actin, and α-tubulin as a loading control. A representative of 4 experiments is shown (left panel). Quantitative analysis of expression levels of VSMC markers (right panels). Different letters indicate significant differences between groups as determined by one-way ANOVA followed by Tukey's test.

Figure S12

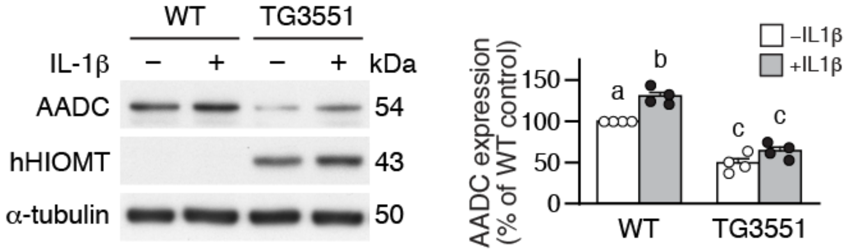

Fig. S12. Overexpression of hHIOMT decreases AADC levels in VSMCs. Primary cultured WT and *hHIOMT* transgenic (line 3551) VSMCs were serum-starved and then treated with or without IL-1β (10 ng/mL). Proteins were prepared 24 h later for Western blotting to detect AADC, hHIOMT, and α-tubulin as a loading control. A representative of 4 experiments is shown. Different letters indicate significant differences between groups as determined by one-way ANOVA followed by Tukey's test.
